# Regional variation in social norm nudges

**DOI:** 10.1038/s41598-024-65765-z

**Published:** 2024-07-22

**Authors:** Sebastian J. Goerg, Andreas Pondorfer, Valentina Stöhr

**Affiliations:** 1 Campus Straubing for Biotechnology and Sustainability, Technical University of Munich, München, Germany; 2 TUM School of Management, Technical University of Munich, München, Germany

**Keywords:** Psychology and behaviour, Climate-change policy

## Abstract

Public support is crucial for the effectiveness of ambitious climate policies, and social norm interventions have been proven effective in fostering support. An open question is which norms should be communicated if support and estimated support for climate policies differ substantially between regions. In two studies, we investigate whether individuals accurately assess the existing support and then explore the impact of national and regional norms on public support. Our results show that the norm on climate policy support is generally misperceived, i.e., the norm is higher than expected. This misperception increases with policy ambition and varies substantially between sub-national regions. Information about the national norm increases support, mainly in regions with below or above-average support. In contrast, interventions with regional norms are ineffective and even backfire in low-support regions. This demonstrates that norm nudges need to consider the regional aspects of the reference and target groups.

## Introduction

Global climate policies are essential to solving the collective action problem related to climate change. Supranational institutions such as the European Union (EU) recently passed large programs (e.g., Fit-for-55) to drastically reduce greenhouse gas emissions by 2030. Such ambitious climate policies are urgently needed to mitigate the consequences of climate change and keep our world sustainable^1,2^. However, strong public support for climate policies is crucial for them to be effective^3–7^. A growing body of research in the behavioral and social sciences highlights the potentially productive role of social norms and norm interventions in changing behavior toward support for more climate change action^8–14^.

To date, a significant but underexplored aspect of norm interventions is the substantial regional variation in behaviors and beliefs both between and within countries^15–17^, as well as the regional differences in the accuracy of beliefs about others' attitudes^18^. Previous studies have documented the wide variation in social norms across traditional societies and modern societies^19–21^ and the effect of norms on behavior is likely to depend on the cultural and economic context^21–25^.

Social norms are defined as the prevailing attitudes and behavior within a social group^26^. A common conceptual distinction exists between descriptive social norms, which denote prevalent attitudes and behaviors within a social group, and injunctive social norms, which signify attitudes and behaviors deemed appropriate by the group^27^. A key difference between the two is that descriptive norms typically lack social sanctions for nonconformity, whereas injunctive norms include such sanctions^27,28^. In this study, we elicit empirical beliefs about other people’s EU climate policy support (study 1) and use this information in norm messages to study how people adapt their own support for large-scale EU climate policies (study 2). Since empirical beliefs involve neither a moral imperative nor social sanctions, they can be more associated with descriptive social norms.

Using two experimental online surveys representative of the German population at the sub-national level, this paper makes three contributions to the literature on social norms and climate policy support (we define estimated support, i.e., the empirical belief about common behavior, as the descriptive social norm). First, we find increasing misperceptions of social norms, i.e., people underestimate others’ support of EU climate policies, even more so when these policies become more ambitious (study 1). Second, we document substantial regional variation in misperceived social norms (study 1). Third, we demonstrate that norm interventions informing about the actual support in society at the national level are more effective in increasing individual support than interventions informing about support at the regional level (study 2). More precisely, we show that national norm interventions increase support only at the extreme ends of the sample population, i.e., where the regional average of support is either below or above the national average. In contrast, regional norm interventions backfire and decrease EU climate policy support in low-support regions.

Study 1, conducted in Fall 2021 shortly after the announcement of Fit-for-55, examines how norm misperceptions evolve with the introduction of new policies, comparing existing EU climate policy with more ambitious Fit-for-55 goals. Changes of legal rules or new policies can shift social norms. For example, civil rights legislation in the US South led to major shifts in attitudes and behaviors regarding racism, racial stereotypes, discrimination, and racial slurs^29,30^. Theoretical literature on social norms^31–33^ suggests that new information should lead to updates in perceptions of social norms. Empirical evidence for this mechanism exists among others in the context of gender norms^34^ or climate change support^35,36^. One might expect individuals to have more accurate beliefs about support for existing policies due to longer observation periods, whereas new policies offer fewer chances to observe others' attitudes or opinions, making it unclear if this leads to misperception of social norms. Study 1 addresses the empirical question whether misperception of social norms increases or decreases and provides new insights significant for climate policies and belief updating when information is not provided about others’ beliefs^34,36^ but about a new large-scale policy.

Overall, our findings contribute to the vast literature in psychology, sociology, and economics that documents the influence of social norms on behavior and preferences^37–41^. Experimental research has shown that interventions raising awareness about social norms increase pro-environmental behavior, such as recycling, energy, and water conservation or sustainable food choices^9,42–44^. Additionally, norms are found to positively affect societal approval of conservation behavior^45^ and climate policies concerning topics such as carbon taxes, green energy, food waste or pollution^46–49^.

While most studies found a positive effect of social norms on pro-environmental behavior and policy support, recent empirical research shows that messages of norm nudges might backfire^50^. For example, when the social norm is not climate-friendly enough, pro-environmental behavior might decrease as behaving in a way that is bad for the environment is seen as socially acceptable ^47,51,52^. Thus, testing social norm nudges before implementing them on a large scale in order to prevent promoting the "wrong" social norm is essential^53–55^. One crucial element of a norm nudge is the identification of the reference group the norm is based on^35,50^. A norm from a group one is (socially) closer to might be more effective than from a more distant group^14,53,56,57^. Further, beliefs and behavior appear to be strongly linked to local social norms^18,58–60^ and even arbitrary norms work better if the recipient identifies more with the group the norm is coming from^61^. Therefore, a regional norm—the regional population being closer—might have more impact than a national norm.

## Study 1: Norms and misperceptions

In this study, we apply an online experiment to test the impact of supranational policies on support and perceived social norms. We apply a within-subject design to study how support changes when respondents receive information about less ambitious emission reduction goals (low goals) and more ambitious emission reduction goals (high goals). Exploiting the representative nature of our dataset at the regional level, we explore regional variation of norms and investigate how accurate perceptions of norms are.

### Results

**Figure 1:**
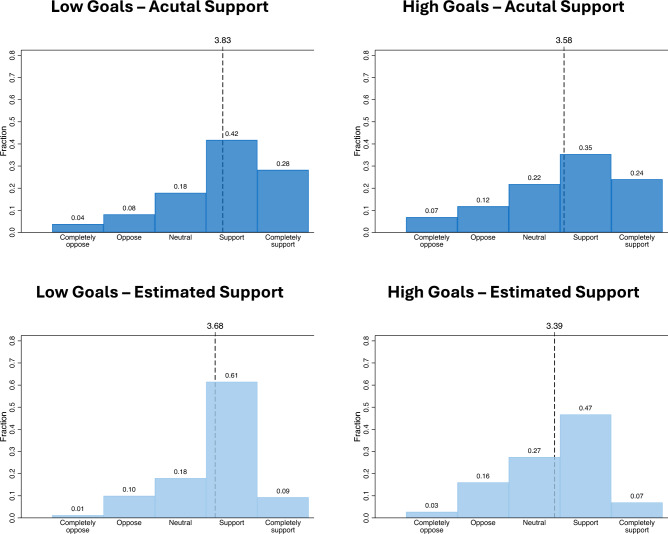
Actual support and estimated support for climate policies under low and high climate goals. The figure shows the distribution of actual and estimated support for low (left) and high (right) climate goals. Both actual and estimated support are measured on a 5-point scale (completely oppose to completely support with neutral option). Each panel indicates average actual support and estimated support as vertical lines (dashed).

Figure 1 shows the distribution of actual and estimated support for low and high EU climate goals (measured on a 5-point scale). For the low goals, the mean actual support is 3.83 and the mean estimated support 3.68 (mean difference (MD) = 0.149, 95% confidence interval (CI) (0.125, 0.172), two-sided t-test = 12.46, p < 0.0001, n = 7,191). This difference corresponds to a decrease of 2.98% on a 5-point scale. Interestingly, the major shift between actual support and estimated support occurs between the support and completely support category. While 28% (42%) of respondents completely support (support) low EU climate goals, 9% (61%) believe others completely support (support) these policies. For the high goals, mean actual support amounts to 3.58 and mean estimated support to 3.39 (MD = 0.186, 95% CI (0.163, 0.209), two-sided t-test = 15.95, p < 0.0001, n = 7,191). This difference corresponds to a decrease of 3.72% on a 5-point scale. Again, the major shift occurs within the positive responses: 24% (35%) of respondents completely support (support) high EU climate goals, while 7% (47%) believe others completely support (support) these policies.

Overall, actual support is higher than estimated support for both low and high climate goals when comparing the average responses of the 5-point-scale. This means that the misperception error (estimated support—actual support) is negative, i.e., respondents generally believe that society is less supportive than it actually is. The difference between actual support and estimated support intensifies with higher climate goals, i.e., misperception becomes more negative with a mean of -0.15 before and -0.19 after the treatment (MD = 0.038, 95% CI (0.019, 0.056), two-sided t-test = 3.96, p = 0.0001, n = 7,191). However, the difference of 0.038 corresponds to a relatively small effect size of about 0.8% on a 5-point scale. When comparing only positive responses (support and completely support) with the remaining categories, a slightly different pattern emerges. For low policy goals, positive responses for actual support and estimated support are identical: 70% of respondents either support or completely support these policies and also believe others do so. In contrast, positive responses between actual support and estimated support are different in high policy goals. 59% of respondents either support or completely support high EU climate goals, while 54% believe others do so. Thus, support is underestimated by 5 percentage points. This difference is significantly different (Pearson chi-squared, p = 0.000, n = 7191).

Next, we test if the results between low goals and high goals are driven by our experimental design. In the control group, respondents received the message of low EU climate goals twice. Repeatedly asking for actual support (1st mean = 3.816, 2nd mean = 3.789, MD = 0.028, 95% CI (− 0.021, 0.076), two-sided t-test = 1.135, p = 0.2587, n = 109) and estimated support (1st mean = 3.844, 2nd mean = 3.807, MD = 0.037, 95% CI (-0.045, 0.118), two-sided t-test = 0.894, p = 0.3735, n = 109) for the low policy goals does not significantly influence responses. All reported findings are robust with Wilcoxon tests and in OLS regressions with additional controls (see Table S2 in SI). Further, beliefs in climate change and positive attitudes towards EU climate policies significantly decrease the gap in the misperception error between treatments (see Figure S2 in SI).

Within this paper, we focus on the descriptive norm, i.e. the norm based on peoples’ actual behavior, instead of the injunctive norm, i.e. the norm based on peoples’ perception of peoples’ actual behavior. We exclude the results for the injunctive norm from the main part of this paper for several reasons: Firstly, research indicated that under cognitive load, the influence of descriptive norms on behavior increases, while the impact of inunctive norms decreases^62^. Others suggest that compliance with injunctive norms often involves strategic considerations related to social status and material benefits, which necessitates greater cognitive effort^63^. Thus, injunctive norms are more difficult to understand and are therefore likely less cost-efficient in actual campaigns. Secondly, results for the injunctive norm prove to be qualitatively the same, only the average magnitude of support is slightly higher. This is in line with a recent meta-study showing that both — injunctive norms and descriptive norms — have an impact on pro-environmental intetions of similar size^64^. Thirdly, the change in misperception error from the low to the high climate goals is larger in the descriptive than the injunctive norm (injunctive norm mean error change = 0.014, descriptive norm mean error change = 0.038, MD = -0.025, 95% CI (-0.044, -0.006), two-sided t-test = -2.53, p = 0.012, n = 7,173). Thus, the descriptive norm apparently leads to a stronger underestimation of society once more ambitious policies are involved. The results for the injunctive norm can be found in Figure S1 in SI. From the sample of 7,191 respondents, 18 did not answer the questions concerning the injunctive norm.

**Figure 2:**
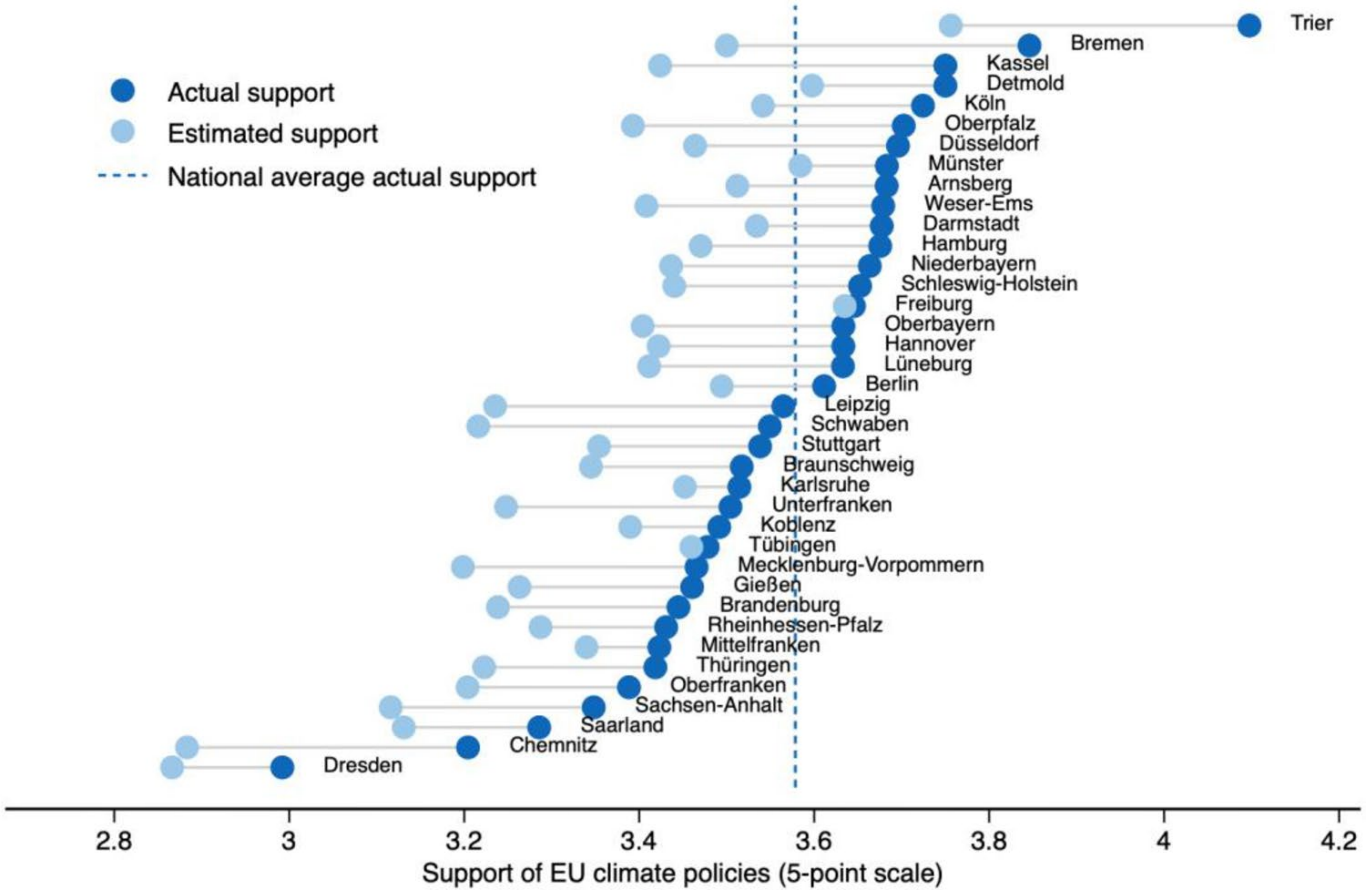
Regional heterogeneity in misperception error for high climate goals. The figure shows mean actual and estimated support for each of the 38 NUTS regions. Both actual and estimated support are measured on a 5-point scale (completely oppose to completely support with neutral option). Here, the misperception error is defined as the difference between mean actual and estimated support for EU climate policies within each respective NUTS2 region. The dashed line represents the national mean of actual support. Observations = 7191.

Figure 2 shows the regional variation in norm misperception for more ambitious EU climate policies (high climate goals), i.e., the difference between mean actual and estimated support across the 38 NUTS2 regions (see Figure S3 in SI for a comparison of the mean misperception error between low and high climate goals). Both actual and estimated support vary substantially between the different regions. Misperception ranges from a mean of -0.01 (0.2% on 5-point scale) in Freiburg to a mean of -0.35 (7% on 5-point scale) in Bremen.

Sorting the regions from lowest to highest regional misperception and testing the quartiles against each other substantiates this finding (1st vs. 2nd quartile: MD = − 0.08, 95% CI (− 0.148, − 0.015), two-sided t-test = − 2.39, p = 0.0168, n = 3,310; 2nd vs. 3rd quartile: MD =− 0.05, 95% CI (− 0.011, − 0.001), two-sided t-test = − 1.91, p = 0.0562, n = 4,528; 3rd vs. 4th quartile: MD = − 0.09, 95% CI (− 0.148, − 0.032), two-sided t-test = − 3.02, p = 0.0025, n = 3,881). These findings are robust in Wilcoxon tests except for the difference between the second and third quartile.

## Study 2: Norm interventions

The first study shows that the social norm of support for climate policies in Germany is misperceived and that the misperception increases with policy ambition. Results also show that support levels and perceived support varies substantially between regions. In this second study, we apply a between-subject design and explore the effect of norm interventions on public support across eight sub-national regions. To do so, we test whether national or regional norm interventions are more effective in increasing support in regions where support is below the national level (n = 3), at the national level (n = 2), and above the national level (n = 3) (see methods section for details).

### Results

**Figure 3:**
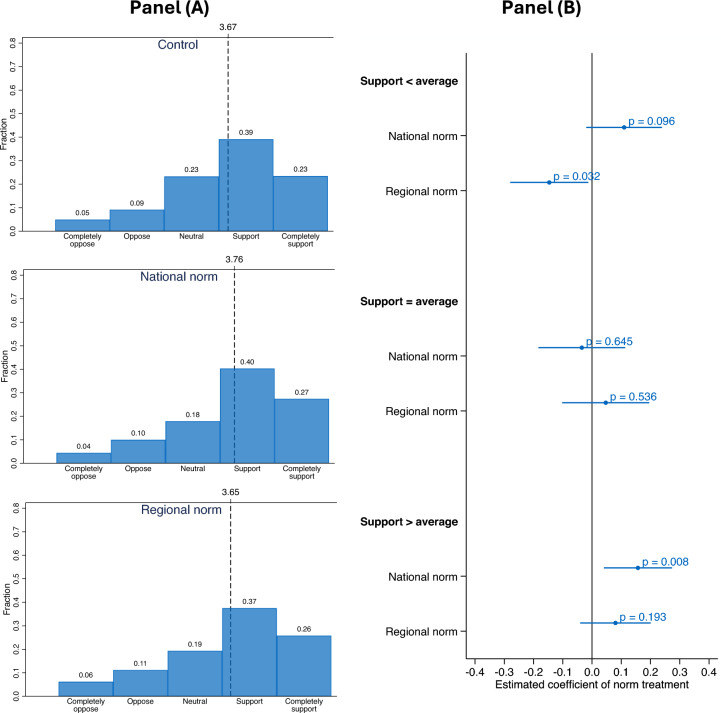
The impact of norm treatments on climate policy support. Panel (**A**) shows the distribution of actual support across control (n = 1,554), national norm treatment (1,591), and regional norm treatment (n = 1.582). Support is measured on a 5-point scale (completely oppose to completely support with neutral option). Each panel indicates average support as vertical lines (dashed). Panel (**B**) shows coefficients based on an Ordinary Least Square (OLS) regression of the pooled data. The dependent variable is support (5-point scale). The explanatory variables are national norm treatment and regional norm treatment and their interaction with sub-national regions (i.e., support below national average, support at national average, and support above national average). To control for potential confounders caused by unobserved heterogeneity across regions, regional fixed effects are included in the specification. Bars indicate 95% confidence intervals obtained from heteroscedasticity robust standard errors.

Figure 3 Panel (A) shows the distribution of support in the control and treatment groups. The patterns of the distributions are comparable to study one. A Spearman correlation indicates a significant relationship between mean support of study two (control group) and mean support of study one (high goals) within the eight sub-national regions (Spearman’s rho = 0.786, p = 0.0208, n = 8).

Regarding treatment effects, Fig. 3 Panel (A) shows that the mean support of 3.76 in the national norm intervention is significantly higher than the mean support of 3.67 in the control group (MD = -0.93, 95% confidence interval (CI) (-0.169, -0.016), two-sided t-test = -2.39, p = 0.017, n = 3,145). This corresponds to an effect size of about 1.9% on a 5-point scale. In contrast, mean support between control and the regional norm intervention are statistically not different (MD = 0.016, 0 95% confidence interval (CI) (-0.063, 0.094), two-sided t-test = 0.39, p = 0.695, n = 3,136).

Next, we investigate treatment effects in regions with different levels of support. To do so, we apply an Ordinary Least Square (OLS) regression with support as the dependent variable and interactions between treatments and regions as explanatory variables. Figure 3 Panel (B) displays the estimated coefficients of this exercise. Zooming into regions, the following heterogeneous patterns emerge. First, regional norm interventions significantly decrease support by 2.92% in regions where support is below the national average (coef. -0.146, p = 0.032, 95% CI (-0.280, -0.014)). Second, both — national and regional norm interventions — have no impact on support in regions where support is at the national average. Third, national norm interventions significantly increase support by 3.14% in regions where support is above the national average (coef. 0.157, p = 0.008, 95% CI (0.040, 0.274)) and weakly significantly increase support by 2.20% in regions where support is below the national (coef. 0.110, p = 0.096, 95% CI (-0.019, 0.241)). Thus, the overall positive effect of national norm interventions is mainly driven by behavioral responses to interventions in regions with low or high support. These findings are robust in Wilcoxon tests and OLS regressions with additional controls such as climate change beliefs, belief in implementation of climate policies, and trust in institutions (see Figure S4 and Table S4 in the appendix).

We also address potential confounders, such as different support levels across regions or different levels in norm messages. First, structural, economic, or cultural specifics may drive differences in support levels across regions. Therefore, controlling for these unobserved characteristics at the regional level is important. To focus on variation within regions and isolate the impact of confounding factors driving differences in support levels, regional fixed effects are included in the estimation of Figure 3 Panel (A). Second, the estimated coefficients of national norm interventions are similar in regions with low and high support (coef. 0.110 vs. coef. 0.157, p = 0.595, 95% CI (-0.221, 0.127)). Since messages of national norm interventions are identical, different levels of support across regions are unlikely to drive results. Third, the design of the study allows us to investigate regional norm messages with different levels. In low (high) support regions, participants received the information that 47% (64%) and 38% (66%) of others support climate policies, respectively. The estimated coefficients of different regional norm messages are statistically not distinguishable (region with support below average: coef. -0.208 vs. coef. -0.115, p = 0.478, 95% CI (-0.349, 0.163); region with support above average: coef. 0.137 vs. coef. 0.051, p = 0.451, 95% CI (-0.137, 0.308), see Figure S5 in the appendix). Finally, if levels of norm messages are important, we would expect that the regional norm intervention displaying the highest level of norm messages outperforms the national norm intervention in high-support regions. This is not the case. The estimated coefficients do not differ significantly, as displayed in Fig. 3 Panel (A) (coef. 0.157 vs. coef. 0.080, p = 0.200, 95% CI (-0.041, 0.195)). Overall, these findings confirm that national norm messages are most effective in increasing support, while regional norm messages decrease support in regions with low average support.

## Discussion and conclusion

Effective implementation of ambitious climate policies relies on strong public support^3–7^. Social norm interventions provide a powerful tool to increase climate-friendly behavior and support for climate policies^9,48,54^. However, the utilization of norms to drive behavioral change requires careful consideration of various caveats, notably the regional dynamics of the reference and target groups and the potential for norm messages to backfire^51,53,54,58,65^. This paper sets out to address these caveats by looking into the variation in people’s perception of others’ support and the effect of national and regional norm interventions on support.

This article shows that respondents’ estimation of others' support is generally lower than the actual average support. This misperception of social norms is in line with recent experiments conducted in the US finding similar results^18,48^. Introducing more ambitious climate policies not only results in lower support but also reduces the estimated support of others even further. Previous studies showed that policy can support social changes^66^ and provide reasons for people to change their expectations^67^ to solve the collective action problem of climate change. The results of this study suggest that the current design of EU climate policies leads to changes in perceived social norms that may have the opposite effect: lack of support and decreasing climate change action.

Further, we show that these perceived social norms vary substantially across sub-national regions in Germany. This indicates that regional norms may deviate strongly from national norms. Keeping in mind that local norms appear to be strongly correlated with peoples’ beliefs and behavior^18,58–60^, such a difference in underlying norms might play a considerable role in the formation of varying beliefs and behavior within a society. To this end, we conducted a second experiment to test the effectiveness of interventions using either national social norms or regional social norms as manipulation. The results reveal that—on average—national norm interventions are particularly effective in increasing support for EU climate policies. Regional norm interventions backfire in low-support regions and decrease support even further. The latter finding is in line with Rinscheid et al.^68^ who find that communicating low support for sustainable policies or behavior in regional norm messages decreases policy support. However, an advantage of our study is that we provide norm messages based on the actual beliefs of the reference group instead of artificial ones. This increases the creditability of norm messages and trust in policymakers that aim to shift support through norm interventions such as information provision campaigns. In line with previous studies utilizing social norm appeals^69^, observed effect sizes are significant but relatively small.

The results of this study have further value for policymakers as national norm interventions may, on average, be effective and cost-efficient but do not change attitudes and behavior among large fractions of the population. Nevertheless, whether our results extend beyond Germany and the context of support for EU policies requires further investigation. Finally, this paper aligns with the literature’s plea to explore the efficacy of social norm nudges^53–55^ and highlights the importance of accounting for regional variations in social norms.

## Methods

Ethical approval was granted by the German Association for Experimental Economic Research (GfeW). The document is available at (https://gfew.de/ethik/Uo1auAot). Methods were carried out in accordance with the guidelines of the GfeW and experimental protocols were approved by them. Informed consent was obtained from all participants in the study.

### Study 1

#### Survey data and sampling

The data for the pre-registered online survey experiment (https://osf.io/bhc2k) was collected from the 24th of August to the 23rd of October 2021. All information and survey questions were presented in German language. Our sample is regionally representative of the resident population aged 18 and older. In particular, respondents are representative for gender and three different income groups (less than 1,500 Euro, 1,500—4,000 Euro, more than 4,000 Euro) across 38 NUTS2 regions. National quotas deviate by less than 0.5%. Quotas on the NUTS2 level deviate by a maximum of 11.8% with a median deviation of less than 1.8% (national and NUTS2 quotas for gender and population in 2020 are based on the statistical office of the European Union (EUROSTAT). National and NUTS2 quotas for income are based on data from the German General Social Survey (ALLBUS) from 2018). Respondents were recruited by the market research institute respondi and the study was conducted using the online surveying platform Qualtrics. One person had to be excluded because she did not finish the questionnaire, and four participants were dropped due to unreasonable age specifications of more than 100 years. Finally, 211 people did not answer at least one of the questions on actual and estimated support. This leaves a total of 7,300 respondents, 109 of whom were part of the control group and 7,191 were in the treatment group.

#### Experimental design

**Figure 4:**
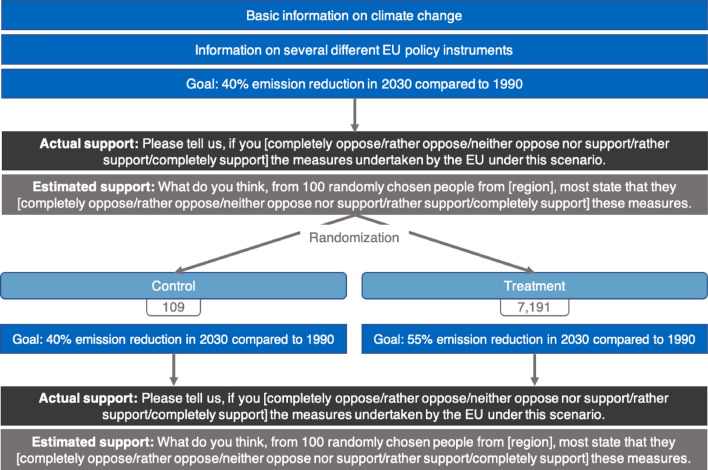
Survey flow.

The experiment is set up as a hypothetical vignette study. Figure 4 provides a summary of the survey experiment. The procedure was as follows. First, all respondents received basic information on the effect of GHG emissions, specifically CO2, on global warming and an average European household’s CO2 emissions. Second, several different EU policy instruments to reduce GHG emissions, i.e., the expansion of renewable energy, investment in energy efficiency, and the EU Emissions Trading System (EU-ETS), were presented. Participants are requested to suppose that the EU plans on reducing GHG emissions by 40% until 2030 compared to 1990 (the EU-ETS is the EU’s GHG emissions trading scheme. It works on the “cap and trade” principle, meaning that a total amount of emittable GHGs is determined, and emission allowances are traded within this cap, resulting in a price for GHG emissions such as CO_2_). After asking for individual support and their estimate of others’ support for the aforementioned measures taken by the EU under this hypothetical low climate goals scenario, participants in the treatment group are requested to assume that the EU now wants to take on more ambitious, i.e., high, climate goals due to the Green Deal meaning that the GHG emission reduction goal is increased to 55% in 2030 compared to 1990. Afterwards, support and estimated support for the EU policy instruments under these new scenarios is elicited once again. For the control group, the initial scenario is repeated. Our control group is used to investigate whether repeatedly eliciting support and estimated support influence the responses. No analyses at the regional level are intended for the control group; therefore, a smaller sample was collected than in the treatment group. Table S1 in SI provides descriptive statistics for the treatment and control groups.

We elicited respondents’ support for EU climate policies on a five-point scale ranging from 1 to 5 (completely oppose to completely support with neutral option). The same scale was used to elicit respondents’ estimates of others’ support for EU climate policies. The question on estimated support was incentivized. Respondents were informed that they can win 100 Euros if they correctly guess the median answer on the five-point scale of 100 randomly drawn respondents for the question on support for EU climate policies. Incentivized questions further motivate respondents to provide their best guess instead of giving a random answer and thus make the results of this measure even more meaningful^70^ Behavioral studies used comparable elicitation methods to measure social norms^38,48,71^.

Further, we collected the following control variables. Recent hazard experience was measured by asking respondents about financial losses, the personal burden they experienced due to the COVID-19 pandemic (five-point scale), and whether they were directly or indirectly affected by the flooding (five-point scale). To measure beliefs in climate change, we construct an index from responses (four-point scale) to 12 statements about climate change. The implementation of climate protection in the EU, Germany, and the respective NUTS2 regions was measured by asking whether climate protection was seriously pursued and implemented in the respective region (four-point scale). Trust in climate-friendly companies and scientists was measured on a four-point scale. The summary index for trust in international (national) institutions is based on two (three) general trust questions related to the EU and UN, respectively (city, state, and national government), also measured on a four-point scale. See SI for more details about individual-level measures.

### Study 2

#### Survey data and sampling

We conducted a pre-registered online survey experiment (https://osf.io/hcjfz) in Germany to elicit the effect of national and regional social norms. Data collection took place from the 19th of July to the 4th of August 2022. All information and survey questions were presented in German. The survey was conducted in eight different NUTS2 regions for which the sample is respectively representative of the resident population aged 18 and older. More specifically, respondents are representative for gender and three different age groups (18–40, 41–60, older than 60). Quotas for gender and age are based on data from Eurostat, the statistical office of the European Union, from 2021. A total of 4,800 respondents were recruited by the market research institute Bilendi, and the study was conducted using the online platform Qualtrics. These 4,800 respondents are made up of 600 respondents per each of the eight NUTS2 regions in which the survey was conducted. Four people had to be dropped since they participated in the survey twice. 69 people opted not to answer the question on support. This leaves a total of 4,727 respondents.

#### Experimental design

**Figure 5:**
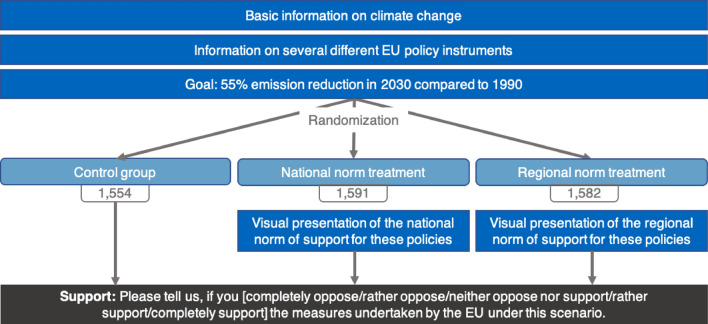
Survey flow.

In this experiment, we use vignettes in which respondents were asked to state their support for EU climate policies employing different national and regional social norms. We specifically selected the three regions with the highest support (Arnsberg, Detmold, Darmstadt), the two with average support (Lüneburg, Brandenburg), and the three with the lowest support (Dresden, Chemnitz, Saarland) from our first study to conduct this second study in (the highest support in study 1 was measured in Trier; however, this region is too small to retrieve a sample of 600 respondents and we excluded it from this survey). For this purpose, support was turned into a dummy variable coded 1 for "rather support" or "completely support" and 0 otherwise. Based on this dummy, the national and regional norms were presented as percentages. Within these regions, respondents are randomly allocated between the national norm treatment, the regional norm treatment, and the control group (see Table S3 in SI for a randomization check across treatments). Figure 5 provides a summary of the survey experiment. Same as in the first study, all respondents initially receive basic information on the effect of GHG emissions, on global warming, and an average European household’s CO2 emissions, as well as information on several different EU policy instruments to reduce GHG emissions. Participants are requested to suppose that the EU plans on reducing GHG emissions by 55% until 2030 compared to 1990, i.e., the ambitious climate policies scenario of the first study. In the next step, respondents in the national norm treatment see a visual presentation of the national norm of support for these policies, measured in our first study. Respondents in the regional norm treatment see the same presentation, however, containing the regional norm of the respective NUTS2 region they are living in. Respondents in the control group do not see any visual representation or indication of a norm. Afterward, respondents are asked to state their support for the aforementioned measures taken by the EU under this hypothetical scenario.

We elicited respondents’ support for EU climate policies on a five-point scale ranging from 1 to 5 (completely opposed to completely supported with neutral option)- We measured respondents’ individual-level characteristics in the same way as in the first study.

### Supplementary Information

Supplementary Information 1.

Supplementary Information 2.

## Data Availability

All data generated or analyzed during this study are included in this published article (and its Supplementary Information files).
