# Supplementary material for: Regional variation in social norm nudges
Source: Sci Rep. 2024 Jul 22;14:16773. doi: 10.1038/s41598-024-65765-z (PMC11263545; doi:10.1038/s41598-024-65765-z)
Supplement: Supplementary file 1 — Supplementary Information 1. [file 41598_2024_65765_MOESM1_ESM.pdf]

Supplementary Information:

Regional variation in social norm nudges

June 16, 2024

Contents

1 Materials and Methods **6**

1.1 Experimental instructions for survey 1 6

1.1.1 Basic information on climate change and EU climate policies (all respondents) 6

1.1.2 Information provision on EU policy instruments (all respondents) . . 6

1.1.3 Introduction of first hypothetical scenario (all respondents) 8

1.1.4 Treatment group: Introduction of second hypothetical scenario 9

1.1.5 Control Group: Introduction of second hypothetical scenario 10

1.1.6 Survey Screenshots in German language 12

1.2 Experimental instructions for survey 2 17

1.2.1 Basic information on climate change and EU climate policies (all respondents) 17

1.2.2 Information provision on EU policy instruments (all respondents) . . 17

1.2.3 Introduction of hypothetical scenario (all respondents) 19

1.2.4 National/regional treatment group: Introduction of [national/regional] norm 19

1.2.5 Survey Screenshots in German language 21

1.3 Wording of survey items and construction of summary indices 25

1.3.1 Socio-demographic characteristics 25

1.3.2 Recent hazards 25

1.3.3 Other factors 26

2 Supplementary Analysis (robustness checks) **29**

2.1 Randomization check 29

2.2 Non-parametric tests 29

2.3 Effect of high climate goals on public support for climate policies 30

2.4 Correlations with individual factors for high climate goals 31
2.5 Treatment effect of social norms on support 32

Tables **33**

Figures **37**

List of Tables

1 Descriptive statistics and randomization check for survey 1 33

2 Effect of high climate goals on public support for climate policies 34

3 Descriptive statistics and randomization check for survey 2 35

4 Treatment effect of social norms on support 36

List of Figures

1 Mean support for climate policies under low and high climate goals for injunctive norm 37
2 Correlations with individual factors for high climate goals 38
3 Regional heterogeneity in misperception error for low and high climate goals 39
4 Regional heterogeneity in misperception error for low and high climate goals 40
5 Norm treatments within regoins 41

1 Materials and Methods

1.1 Experimental instructions for survey 1

The following paragraphs outline the experimental instructions as shown to participants. Instructions have been translated from German. Screenshots of the original instructions are shown in Section SI1.1.6.

1.1.1 Basic information on climate change and EU climate policies (all respondents)

Information about climate change

Since the beginning of industrialization people have been emitting large amounts of greenhouse gases, for example by burning coal, oil, and gas. An example for greenhouse gases is carbon dioxide (CO2). These greenhouse gases cause a gradual increase of the average global temperature. Since 1900, the earth's temperature has risen around 1°C.

The average European household produces around 15.5 tons of CO2 per year. This indicator is known as carbon footprint or ecological footprint.

Further developments depend in particular on the amount of greenhouse gases being emitted in the future. If the current trend continues, the average global temperature is likely to increase by up to 3°C by the end of this century.

1.1.2 Information provision on EU policy instruments (all respondents)

Information about European Union politics

To curb the consequences of climate change the European Union (EU) plans to reduce the emission of greenhouse gases. Despite the current COVID-19 pandemic, the EU wants to stick to their climate targets.

To reduce greenhouse gases, the EU relies on the following measures:

<u>Expansion of renewable energies</u>

Sustainable climate policy should further expand bioenergy, geothermal energy, hydropower, ocean energy, solar energy, and wind energy.

<u>Increase of energy efficiency</u>

Energy efficiency should be increased in the following areas: i) public and private transport, ii) energy efficient buildings and in iii) industrial processes.

<u>Expansion of emissions trading</u>

Emissions trading requires the presentation of a valid emission allowance for each ton of CO2 emitted by a group of greenhouse gas producers. The EU determines how many tons of CO2 may be emitted by this group in total. These emission certificates can be bought via emissions trading. If CO2 is emitted without a certificate, penalty payments are required. Emitting little CO2 leads correspondingly to spending little on certificates. **A reduction in the amount of emission certificates usually results in a higher price per ton of CO2 emitted and thus increases the costs for greenhouse gas producers.**

The EU Emissions Trading System:

- includes 30 European countries and covers around 40% of the greenhouse gas emissions in the EU.
- limits emissions from around 11.000 plants in the energy sector and in the manufac-

turing industry as well as emissions from air carriers.

- **should also consider emissions from housing and transport in the future and can therefore affect the prices of fossil fuels (e.g. heating oil) and fuels (e.g. petrol and diesel).**

Current trend of greenhouse gases

In this figure you can see the development of greenhouse gas emissions (in million tons of CO2) in the EU from 1990 to 2020. The figure shows that by 2020 already 20% less greenhouse gases have been emitted than in 1990.

Now, the EU plans to further reduce greenhouse gas emissions until 2030. In the figure, this year is marked with a red line.

Figure: Development of greenhouse gas emissions

1.1.3 Introduction of first hypothetical scenario (all respondents)

Now we ask about your opinion on EU climate policy.

As a reminder:

- The expansion of renewable energies, the increase in energy efficiency and the expansion of emissions trading are key measures of EU climate policy.
- A reduction in the amount of emission certificates through EU policies usually results in a **higher price per ton of CO2 and in higher costs for greenhouse gas producers.**
- The average European household produces around 15.5 tons of CO2 per year.

Please consider the following hypothetical scenario:

The EU plans to **reduce greenhouse gases by up to 40%** until 2030 compared to 1990 (see figure). Assume that besides the industry, households are also influenced by the measures.

Other countries outside the EU (e.g. China, USA) are pursuing climate targets to reduce emissions as well.

Figure: Reduction of greenhouse gas emissions by 40% until 2030

1.1.4 Treatment group: Introduction of second hypothetical scenario

Now we ask about your opinion on a <u>changed</u> EU climate policy.

As a reminder:

- The expansion of renewable energies, the increase in energy efficiency and the expansion of emissions trading are key measures of EU climate policy.
- A reduction in the amount of emission certificates through EU policies usually results in a **higher price per ton of CO2 and in higher costs for greenhouse gas producers.**
- The average European household produces around 15.5 tons of CO2 per year.

Please consider the following hypothetical scenario:

With the European 'Green Deal', the EU wants to create a more ambitious climate target. Therefore, the EU plans to **reduce greenhouse gas emissions** in 2030 **by up to 55%** instead of 40% compared to 1990 (see figure). Assume that besides the industry, households are also influenced by the measures.

Other countries outside the EU (e.g. China, USA) are pursuing climate targets to reduce emissions as well.

Figure: Reduction of greenhouse gas emissions by 40% vs 55% until 2030

1.1.5 Control Group: Introduction of second hypothetical scenario

Now we ask about your opinion on EU climate policy again.

You get to see the same information again. This is for verification of the data quality and helps to better understand your answers. It is not an error.

As a reminder:

- The expansion of renewable energies, the increase in energy efficiency and the expansion of emissions trading are key measures of EU climate policy.
- A reduction in the amount of emission certificates through EU policies usually results in a **higher price per ton of CO2 and in higher costs for greenhouse gas producers.**
- The average European household produces around 15.5 tons of CO2 per year.

Please consider the following hypothetical scenario:

The EU plans to **reduce greenhouse gases by up to 40%** until 2030 compared to 1990 (see figure). Assume that besides the industry, households are also influenced by the measures.

Other countries outside the EU (e.g. China, USA) are pursuing climate targets to reduce emissions as well.

Figure: Reduction of greenhouse gas emissions by 40% until 2030

1.1.6 Survey Screenshots in German language

Screen 1

Nun folgen einige einführende Informationen zum Klimawandel und der Klimapolitik der Europäischen Union.

Bevor Sie im Folgenden zu Ihrer Meinung befragt werden, bitten wir Sie, die folgenden Informationen über den Klimawandel und die Klimapolitik der Europäischen Union aufmerksam durchzulesen.

→

Screen 2

Informationen zum Klimawandel

Seit dem Beginn der Industrialisierung stoßen Menschen beispielsweise durch das Verbrennen von Kohle, Öl und Gas große Mengen von Treibhausgasen aus. Ein Beispiel für Treibhausgase ist Kohlendioxid (CO_2). Diese Treibhausgase bewirken, dass die durchschnittliche Erdtemperatur allmählich ansteigt. Seit 1900 ist die Erdtemperatur im Durchschnitt um etwa 1°C gestiegen.

Der durchschnittliche Haushalt in Europa produziert ca. 15,5 Tonnen CO_2 pro Jahr. Dieser Indikator wird auch als CO_2-Fußabdruck oder ökologischer Fußabdruck bezeichnet.

Die weitere Entwicklung hängt insbesondere davon ab, ob in Zukunft wenig oder viel Treibhausgase ausgestoßen werden. Wenn der derzeitige Trend anhält, steigt die durchschnittliche Erdtemperatur bis Ende dieses Jahrhunderts wahrscheinlich um bis zu 3°C an.

→

Screen 3

Informationen zur Politik der Europäischen Union

Um die Folgen des Klimawandels einzudämmen, plant die Europäische Union (EU) den Ausstoß von Treibhausgasen zu senken. Trotz der aktuellen Corona-Pandemie will die EU an ihren Klimazielen festhalten.

Zur Reduktion von Treibhausgasen setzt die EU auf folgende Maßnahmen:

Ausbau erneuerbarer Energien

Eine nachhaltige Energiepolitik soll Bioenergie, Geothermie, Wasserkraft, Meeresenergie, Sonnenenergie und Windenergie weiter ausbauen.

Steigerung der Energieeffizienz

Die Energieeffizienz soll in den folgenden Bereichen gesteigert werden: i) öffentlicher und privater Verkehr, ii) energieeffiziente Gebäude und bei iii) industriellen Verfahren.

Ausbau des Emissionshandels

Beim Emissionshandel muss eine Gruppe von Treibhausgasproduzenten für jede ausgestoßene Tonne an CO_2 eine gültige Emissionsberechtigung vorlegen. Die EU legt fest, wie viele Tonnen CO_2 von dieser Gruppe insgesamt ausgestoßen werden dürfen. Diese Emissionsberechtigungen können im Emissionshandel gekauft werden. Wird ohne Berechtigung CO_2 emittiert, sind Strafzahlungen fällig. Wer wenig CO_2 emittiert, muss entsprechend wenig für Berechtigungen ausgeben. **Eine Verringerung der Menge an Emissionsberechtigungen führt in der Regel zu einem höheren Preis pro ausgestoßener Tonne CO_2 und erhöht damit die Kosten für Treibhausgasproduzenten.**

Das EU-Emissionshandelssystem:

- umfasst 30 europäische Länder und deckt ca. 40% der Treibhausgasemissionen in der EU ab.
- begrenzt die Emissionen von rund 11.000 Anlagen im Stromsektor und in der verarbeitenden Industrie sowie die Emissionen von Luftfahrtunternehmen.
- **soll in Zukunft auch Emissionen aus Wohnen und Verkehr berücksichtigen und kann somit die Preise von fossilen Brennstoffen (z.B. Heizöl) und Kraftstoffen (z.B. Benzin und Diesel) beeinflussen.**

→

Screen 4

Aktueller Trend von Treibhausgasen

In dieser Abbildung sehen Sie die Entwicklung des Ausstoßes von Treibhausgasen (in Millionen Tonnen CO_2) in der EU von 1990 bis 2020. Aus der Abbildung wir ersichtlich, dass im Jahr 2020 bereits etwa 20% weniger Treibhausgase ausgestoßen wurden als im Jahr 1990.

Die EU plant, nun den Ausstoß von Treibhausgasen bis in das Jahr 2030 weiter zu verringern. In der Abbildung ist dieses Jahr mit einer roten Linie gekennzeichnet.

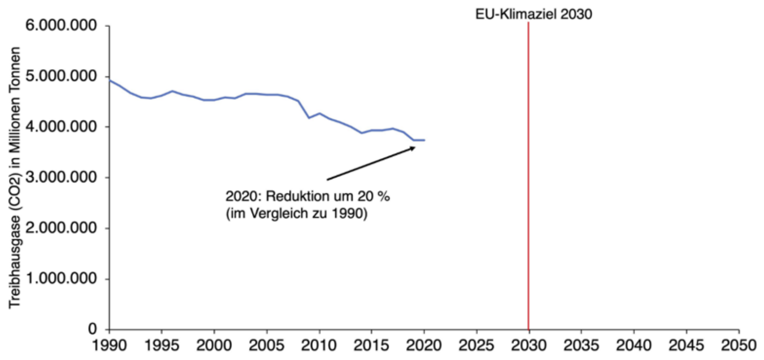

→

Screen 5

Nun fragen wir Sie nach Ihrer Meinung zur EU Klimapolitik.

Zur Erinnerung:

- Der Ausbau der erneuerbaren Energien, die Steigerung der Energieeffizienz und der Ausbau des Emissionshandels sind zentrale Maßnahmen der EU Klimapolitik.
- Eine Verringerung von Emissionsberechtigungen durch die EU-Politik führt in der Regel zu einem **höheren Preis pro Tonne CO_2 und zu höheren Kosten für Treibhausgasproduzenten.**
- Der durchschnittliche Haushalt in Europa produziert ca. 15,5 Tonnen CO_2 pro Jahr.

→

Screen 6

Bitte nehmen Sie folgendes hypothetisches Szenario an:

Die EU plant im Jahr 2030 **die Treibhausgase** im Vergleich zum Jahr 1990 **um bis zu 40% zu senken** (siehe Abbildung). Nehmen Sie an, dass neben der Industrie auch die Haushalte durch die Maßnahmen beeinflusst werden.

Andere Länder außerhalb der EU (z.B. China, USA) verfolgen ebenfalls Klimaziele zur Reduktion von Emissionen.

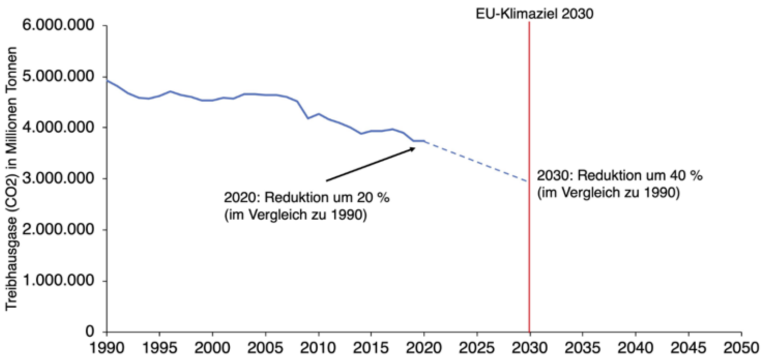

→

Screen 7

Nun fragen wir Sie nach Ihrer Meinung zu einer veränderten EU Klimapolitik.

Zur Erinnerung:

- Der Ausbau der erneuerbaren Energien, die Steigerung der Energieeffizienz und der Ausbau des Emissionshandels sind zentrale Maßnahmen der EU Klimapolitik.
- Eine Verringerung von Emissionsberechtigungen durch die EU-Politik führt in der Regel zu einem **höheren Preis pro Tonne CO_2 und zu höheren Kosten für Treibhausgasproduzenten.**
- Der durchschnittliche Haushalt in Europa produziert ca. 15,5 Tonnen CO_2 pro Jahr.

→

Screen 8

Bitte nehmen Sie folgendes hypothetisches Szenario an:

Mit dem europäischen „Green Deal" will die EU das Klimaziel ambitionierter gestalten. Das heißt, die EU plant im Jahr 2030 **die Treibhausgase** im Vergleich zum Jahr 1990, anstatt um 40%, **um bis zu 55% zu senken** (siehe Abbildung). Nehmen Sie an, dass neben der Industrie auch die Haushalte durch die Maßnahmen beeinflusst werden.

Andere Länder außerhalb der EU (z.B. China, USA) verfolgen ebenfalls Klimaziele zur Reduktion von Emissionen.

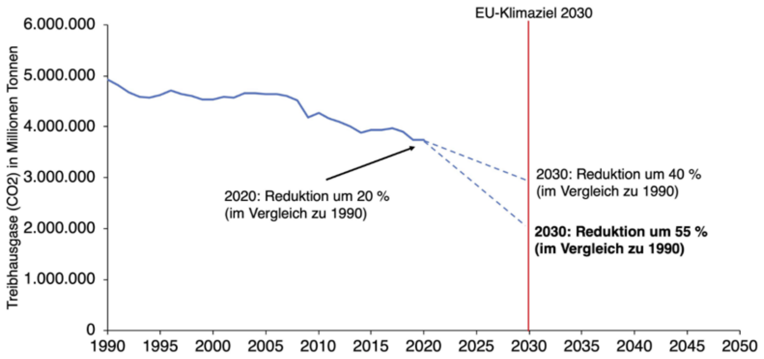

→

1.2 Experimental instructions for survey 2

The following paragraphs outline the experimental instructions as shown to participants. Instructions have been translated from German. Screenshots of the original instructions are shown in Section SI1.2.5.

1.2.1 Basic information on climate change and EU climate policies (all respondents)

Information about climate change

Since the beginning of industrialization people have been emitting large amounts of greenhouse gases, for example by burning coal, oil, and gas. An example for greenhouse gases is carbon dioxide (CO2). These greenhouse gases cause a gradual increase of the average global temperature. Since 1900, the earth's temperature has risen around 1°C.

The average European household produces around 15.5 tons of CO2 per year. This indicator is known as carbon footprint or ecological footprint.

Further developments depend in particular on the amount of greenhouse gases being emitted in the future. If the current trend continues, the average global temperature is likely to increase by up to 3°C by the end of this century.

1.2.2 Information provision on EU policy instruments (all respondents)

Information about European Union politics

To curb the consequences of climate change the European Union (EU) plans to reduce the emission of greenhouse gases. Despite the current COVID-19 pandemic, the EU wants to stick to their climate targets.

To reduce greenhouse gases, the EU relies on the following measures:

Expansion of renewable energies

Sustainable climate policy should further expand bioenergy, geothermal energy, hydropower, ocean energy, solar energy, and wind energy.

Increase of energy efficiency

Energy efficiency should be increased in the following areas: i) public and private transport, ii) energy efficient buildings and in iii) industrial processes.

Expansion of emissions trading

Emissions trading requires the presentation of a valid emission allowance for each ton of CO2 emitted by a group of greenhouse gas producers. The EU determines how many tons of CO2 may be emitted by this group in total. These emission certificates can be bought via emissions trading. If CO2 is emitted without a certificate, penalty payments are required. Emitting little CO2 leads correspondingly to spending little on certificates. **A reduction in the amount of emission certificates usually results in a higher price per ton of CO2 emitted and thus increases the costs for greenhouse gas producers.**

The EU Emissions Trading System:

- includes 30 European countries and covers around 40% of the greenhouse gas emissions in the EU.
- limits emissions from around 11.000 plants in the energy sector and in the manufacturing industry as well as emissions from air carriers.

- **should also consider emissions from housing and transport in the future and can therefore affect the prices of fossil fuels (e.g. heating oil) and fuels (e.g. petrol and diesel).**

1.2.3 Introduction of hypothetical scenario (all respondents)

Please consider the following hypothetical scenario:

In this figure you can see the development of greenhouse gas emissions (in million tons of CO2) in the EU from 1990 to 2020. The figure shows that by 2020 already 20% less greenhouse gases have been emitted than in 1990.

Until now, the EU planed to reduce greenhouse gases by up to 40% until 2030 compared to 1990. In the figure, this year is marked with a red line.

With the European 'Green Deal', the EU wants to create a more ambitious climate target. Therefore, the EU plans to **reduce greenhouse gas emissions** in 2030 **by up to 55%** instead of 40% compared to 1990 (see figure). Assume that besides the industry, households are also influenced by the measures.

Other countries outside the EU (e.g. China, USA) are pursuing climate targets to reduce emissions as well.

Figure: Reduction of greenhouse gas emissions by 40% vs 55% until 2030

1.2.4 National/regional treatment group: Introduction of [national/regional] norm

What does the population in [Germany/region] think about that?

We recently asked [number of respondents in Germany/region] people in [Germany/region]

how they evaluate the measures of the EU under this scenario (55% emission reduction). The participants come from all parts of the population and their answers are representative of the views and stances of the population in [Germany/region]. On the next page you find out how they responded. Please read the information carefully.

Afterwards you are asked about your own opinion.

We asked [number of respondents in Germany/region] people in [Germany/region] how they evaluate the measures of the EU under this scenario (55% emission reduction).

Possible answers included: Completely oppose, rather oppose, neither oppose nor support, rather support, completely support.

Here is the result:

Figure: Percentage of people in [Germany/region] rather or completely supporting these measures

1.2.5 Survey Screenshots in German language

Screen 1

Nun folgen einige einführende Informationen zum Klimawandel und der Klimapolitik der Europäischen Union.

Bevor Sie im Folgenden zu Ihrer Meinung befragt werden, bitten wir Sie, die folgenden Informationen über den Klimawandel und die Klimapolitik der Europäischen Union aufmerksam durchzulesen.

→

Screen2

Informationen zum Klimawandel

Seit dem Beginn der Industrialisierung stoßen Menschen beispielsweise durch das Verbrennen von Kohle, Öl und Gas große Mengen von Treibhausgasen aus. Ein Beispiel für Treibhausgase ist Kohlendioxid (CO_2). Diese Treibhausgase bewirken, dass die durchschnittliche Erdtemperatur allmählich ansteigt. Seit 1900 ist die Erdtemperatur im Durchschnitt um etwa 1°C gestiegen.

Der durchschnittliche Haushalt in Europa produziert ca. 15,5 Tonnen CO_2 pro Jahr. Dieser Indikator wird auch als CO_2-Fußabdruck oder ökologischer Fußabdruck bezeichnet.

Die weitere Entwicklung hängt insbesondere davon ab, ob in Zukunft wenig oder viel Treibhausgase ausgestoßen werden. Wenn der derzeitige Trend anhält, steigt die durchschnittliche Erdtemperatur bis Ende dieses Jahrhunderts wahrscheinlich um bis zu 3°C an.

→

Screen 3

Informationen zur Politik der Europäischen Union

Um die Folgen des Klimawandels einzudämmen, plant die Europäische Union (EU) den Ausstoß von Treibhausgasen zu senken. Trotz der aktuellen Corona-Pandemie will die EU an ihren Klimazielen festhalten.

Zur Reduktion von Treibhausgasen setzt die EU auf folgende Maßnahmen:

Ausbau erneuerbarer Energien

Eine nachhaltige Energiepolitik soll Bioenergie, Geothermie, Wasserkraft, Meeresenergie, Sonnenenergie und Windenergie weiter ausbauen.

Steigerung der Energieeffizienz

Die Energieeffizienz soll in den folgenden Bereichen gesteigert werden: i) öffentlicher und privater Verkehr, ii) energieeffiziente Gebäude und bei iii) industriellen Verfahren.

Ausbau des Emissionshandels

Beim Emissionshandel muss eine Gruppe von Treibhausgasproduzenten für jede ausgestoßene Tonne an CO_2 eine gültige Emissionsberechtigung vorlegen. Die EU legt fest, wie viele Tonnen CO_2 von dieser Gruppe insgesamt ausgestoßen werden dürfen. Diese Emissionsberechtigungen können im Emissionshandel gekauft werden. Wird ohne Berechtigung CO_2 emittiert, sind Strafzahlungen fällig. Wer wenig CO_2 emittiert, muss entsprechend wenig für Berechtigungen ausgeben. **Eine Verringerung der Menge an Emissionsberechtigungen führt in der Regel zu einem höheren Preis pro ausgestoßener Tonne CO_2 und erhöht damit die Kosten für Treibhausgasproduzenten.**

Das EU-Emissionshandelssystem:

- umfasst 30 europäische Länder und deckt ca. 40% der Treibhausgasemissionen in der EU ab.
- begrenzt die Emissionen von rund 11.000 Anlagen im Stromsektor und in der verarbeitenden Industrie sowie die Emissionen von Luftfahrtunternehmen.
- **soll in Zukunft auch Emissionen aus Wohnen und Verkehr berücksichtigen und kann somit die Preise von fossilen Brennstoffen (z.B. Heizöl) und Kraftstoffen (z.B. Benzin und Diesel) beeinflussen.**

→

Screen 4

Bitte nehmen Sie folgendes hypothetisches Szenario an:

In dieser Abbildung sehen Sie die Entwicklung des Ausstoßes von Treibhausgasen (in Millionen Tonnen CO_2) in der EU von 1990 bis 2020. Aus der Abbildung wir ersichtlich, dass im Jahr 2020 bereits etwa 20% weniger Treibhausgase ausgestoßen wurden als im Jahr 1990.

Die EU plante bisher, im Jahr 2030 die Treibhausgase im Vergleich zum Jahr 1990 um bis zu 40% zu senken. In der Abbildung ist dieses Jahr mit einer roten Linie gekennzeichnet.

Mit dem europäischen „Green Deal" will die EU das Klimaziel nun ambitionierter gestalten. Das heißt, die EU plant im Jahr 2030 **die Treibhausgase** im Vergleich zum Jahr 1990, anstatt um 40%, **um bis zu 55% zu senken** (siehe Abbildung). Nehmen Sie an, dass neben der Industrie auch die Haushalte durch die Maßnahmen beeinflusst werden.

Andere Länder außerhalb der EU (z.B. China, USA) verfolgen ebenfalls Klimaziele zur Reduktion von Emissionen.

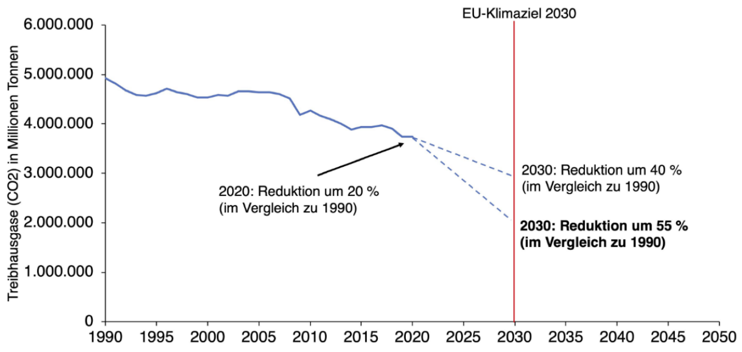

→

Screen 5

Wie sieht das die Bevölkerung in Deutschland?

Wir haben kürzlich 7401 Personen in Deutschland gefragt, wie Sie die Maßnahmen der EU unter diesem Szenario (55% Reduktion von Treibhausgasen) einschätzen. Die Teilnehmenden stammen aus allen Teilen der Bevölkerung und Ihre Antworten repräsentieren die Ansichten und Einstellungen der Bevölkerung in Deutschland. Auf der nächsten Seite werden Sie erfahren, wie sie geantwortet haben. Bitte lesen Sie die Informationen aufmerksam.

Anschließend werden Sie selbst zu Ihrer Meinung befragt.

→

Screen 6

Wir haben 7401 Personen in Deutschland gefragt, wie Sie die Maßnahmen der EU unter diesem Szenario (55% Reduktion von Treibhausgasen) einschätzen.

Die Antwortmöglichkeiten waren: voll und ganz ablehnen, eher ablehnen, weder ablehnen noch unterstützen, eher unterstützen und voll und ganz unterstützen.

Hier ist das Ergebnis:

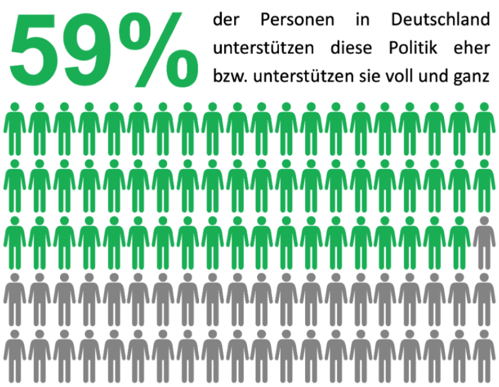

Bitte teilen Sie uns mit, ob Sie die Maßnahmen der EU unter diesem Szenario (55% Reduktion von Treibhausgasen)...

Voll und ganz ablehnen	Eher ablehnen	Weder ablehnen noch unterstützen	Eher unterstützen	Voll und ganz unterstützen	Keine Angabe

→

1.3 Wording of survey items and construction of summary indices

1.3.1 Socio-demographic characteristics

Female: Dummy variable that is coded as 1 if the respondent stated "female" as their gender and 0 otherwise.

Diverse: Dummy variable that is coded as 1 if the respondent stated "diverse" as their gender and 0 otherwise.

Age (median): Dummy variable that is coded as 1 if the respondent is above median age and 0 otherwise.

Income (median): Dummy variable that is coded as 1 if the respondent earns above median income and 0 otherwise.

Education level (tertiary): Dummy variable that is coded as 1 if the respondent has at least a university degree and 0 otherwise.

1.3.2 Recent hazards

Personal burden of COVID-19: "*All in all, how has your household been coping with the COVID-pandemic so far?*" Measured on 5-point scale from 1 to 5 where 1 means "no" and 5 means "very much so".

Financial impact of COVID-19: "*Did you experience any financial losses regarding your salary or otherwise in connection with the COVID-19 pandemic?*" Measured on 5-point scale from 1 to 5 where 1 means "no" and 5 means "very much so".

Impact of recent flood event: "*Have you been or are you directly or indirectly affected by the flood catastrophe that took place in some regions in Germany in July of 2021?*" Measured on 5-point scale from 0 to 4 where 0 means "not at all" and 4 means "very much so".

1.3.3 Other factors

Belief in climate change: Standardized sum of the opinion on 12 statements about climate change, each measured on a 4-point scale where 1 means "completely disagree" and 4 means "completely agree". The higher the score, the more the respondent beliefs in and worries about climate change. The statements are:

1. *"I am concerned about climate change."*
2. *"The consequences of climate change can cause great harm to people in the EU."*
3. *"It is important that the EU climate goal is met."*
4. *"If we act in unison, it is possible to attain the EU climate goal."*
5. *"The actions of a single person have an impact on climate change."*
6. *"Humankind is responsible for climate change."*
7. *"Scientific predictions of climate change are trustworthy."*
8. *"There is a great deal of disagreement among scientists about whether climate change is actually happening."* (Reversely coded)
9. *"I am sure that climate change exists."*
10. *"Climate change is exaggerated in the media."* (Reversely coded)
11. *"Our children should be learning about the causes, effects and potential solutions of global warming in school."*

12. *"There is a link between global warming from greenhouse gas emissions and the more frequent occurrence of extreme weather events such as heavy rainfall."*

Implementation of climate protection in EU: *"Climate protection is seriously persued and effectively implemented in the EU."* Agreement with this statement is measured on a 4-point scale from 1 to 4 where 1 means "completely disagree" and 4 means "completely agree".

Implementation of climate protection in Germany: *"Climate protection is seriously persued and effectively implemented in Germany."* Agreement with this statement is measured on a 4-point scale from 1 to 4 where 1 means "completely disagree" and 4 means "completely agree".

Implementation of climate protection in region: *"Climate protection is seriously persued and effectively implemented in [region]."* Agreement with this statement is measured on a 4-point scale from 1 to 4 where 1 means "completely disagree" and 4 means "completely agree".

Trust in climate friendly companies: *"Please indicate how much you trust the following institutions." ... "companies that invest in climate-protection projects"* Measured on 4-point scale where 1 means "completely distrust" and 4 means "completely trust".

Trust in scientists: *"Please indicate how much you trust the following institutions." ... "scientists that investigate climate change at public research institutions"* Measured on 4-point scale where 1 means "completely distrust" and 4 means "completely trust".

Trust in supranational institutions: Standardized sum of the answer to two questions on trust in institutions, i.e. the UN and EU. Both are measured on a 4-point scale where 1 means "completely distrust" and 4 means "completely trust".

Trust in national institutions: Standardized sum of the answer to three questions on trust in institutions, i.e. the city, state and national government. All three are measured on a 4-point scale where 1 means “completely distrust” and 4 means “completely trust”.

2 Supplementary Analysis (robustness checks)

This section describes the details of the supplementary analysis. The main purpose of the supplementary analysis is to test against potential confounders that may affect our results.

2.1 Randomization check

Tables S1 and S3 show summary statistics across treatments. The last column includes p-values for the null hypothesis that socio-demograhpic characteristics are different across treatments. The null hypothesis can be rejected at conventional levels of statistical significance ($p < 0.05$) for most demographics.

2.2 Non-parametric tests

For both low and high climate goals, the difference between actual and estimated support is significant in non-parametric tests (low goals: Wilcoxon matched-pairs signed-rank test, z =16.04, p = 0.0001, n = 7,191; high goals: Wilcoxon matched-pairs signed-rank test, z = 18.95, p = 0.0001, n = 7,191). The increase in misperception from low to high climate goals is also robust in non-parametric testing (Wilcoxon matched-pairs signed-rank test, z = -2.05, p = 0.0407, n = 7,191).

Looking at regional heterogeneity, except for the difference between the second and thrid quartile the difference between the quartiles of regional misperception remains significant in non-parametric tests (1st vs. 2nd quartile: two sample Wilcoxon rank-sum test, z =-3.127, p = 0.0018, n = 3,310; 2nd vs. 3rd quartile: two sample Wilcoxon rank-sum test, z = 0.835, p = 0.4038, n = 4,528; 3rd vs. 4th quartile: two sample Wilcoxon rank-sum test, z = -6.089, $p < 0.0000$, n = 3,881).

Turning to study 2, the difference between control group means from regions below, at and

above the national average support remains at least weakly significant in non-parametric tests (below vs. at national average: two sample Wilcoxon rank-sum test, z =-1.677, p = 0.0935, n = 966; at vs. above national average: two sample Wilcoxon rank-sum test, z = -2.378, p = 0.0174, n = 978).

The overall effect of the national norm compared to the control group is also robust in non-parametric tests, while the regional norm effect remains insignificant (control vs. national norm: two sample Wilcoxon rank-sum test, z =-2.851, p = 0.0044, n = 3,145; control vs. regional norm: two sample Wilcoxon rank-sum test, z = -0.310, p = 0.7565, n = 3,136). Looking at the split sample, again findings from the t-tests are confirmed by at least weakly significant results (control vs. regional norm in regions with support below the average: two sample Wilcoxon rank-sum test, z =1.845, p = 0.0651, n = 1,177; control vs. national norm in regions with support below the average: two sample Wilcoxon rank-sum test, z =-1.946, p = 0.0517, n = 1,171; control vs. national norm in regions with support above the average: two sample Wilcoxon rank-sum test, z =-2.788, p = 0.0053, n = 1,190).

2.3 Effect of high climate goals on public support for climate policies

We investigate treatment effects of our information provision experiment by conducting regression analysis. Our within-subject design allows us to model the data as a panel. The statistical model underlying the results in Table S2 is

$$y_{ir} = \alpha + \beta ClimatePolicyScenario_i + \gamma' x_{ir} + \epsilon_{ir} \tag{1}$$

where y_{ir} is either the actual or estimated support for climate policies or the misperception error, i.e. the difference between the individual estimated and the average regional actual support, by individual i living in region r. *ClimatePolicyScenario* is a dummy variable which

takes on the value 0 for information about low climate goals and 1 for information about high climate goals. Thus, the coefficient β represents the treatment effect of information about high climate goals on individual support. Note that the low climate goals scenario was repeated in the control group. The constant α represents the mean support for the low goals scenario. x_{ir} is a vector of control variables. It includes socio-demographic characteristics (gender (two dummy variables representing female and diverse with male being the omitted category), age (indicator variable for above-median values), income (indicator variable for above-median values), education level (indicator variable for tertiary education)), NUTS2 regional fixed effects and survey week fixed effects. Regional fixed effects and survey week fixed effects control for omitted variable bias that is specific to regions or the interview time. Standard errors are clustered at the regional NUTS2. The regressions are done separately for the control and treatment group. Finally, for both the regression with the misperception error is repeated with additional controls that can be seen in Table S2.

2.4 Correlations with individual factors for high climate goals

In Figure S2 we show the correlations of individual factors with the absolute value of the misperception error. The statistical model underlying the results in Figure S2 is

$$|MisperceptionError|_{ir} = \alpha + \beta' RecentHazards_i + \delta' OtherFactors_i + \gamma' x_{ir} + \epsilon_{ir} \qquad (2)$$

where $|MisperceptionError|_{ir}$ is the absolute value of the misperception error, i.e. the difference between the individual estimated and the average regional actual support, by individual i living in region r. $RecentHazards_i$ and $OtherFactors_i$ are vectors of the measures listed in Figure S2. x_{ir} is a vector that includes the following control variables: gender (two dummy variables representing female and diverse with male being the omitted category), age (indicator variable for above-median values), income (indicator variable for above-median values), education level (indicator variable for tertiary education), NUTS2 regional fixed effects and

survey week fixed effects. Furthermore, we standardized all explanatory variables to have a mean of zero and a standard deviation of one (z-score), so the coefficients of standardized variables can be interpreted as the change in supporting rates associated with a one standard deviation change in the explanatory variable. Standard errors are clustered at the regional NUTS2 level.

2.5 Treatment effect of social norms on support

Table S4 shows the treatment effect of the national and regional norm treatment on support. The statistical model underlying the results is

$$Support_i = \alpha + \beta\, TreatmentSupportRegion_i + \gamma' x_i + \epsilon_i \tag{3}$$

where $Support_i$ is the support for climate policies by individual i. *TreatmentSupportRegion* is a set of dummy variables representing the interaction between norm treatments and support regions (i.e., national norm x below average support; regional norm x below average support; national norm x at average support; regional norm x average support; national norm x above average support; regional support x above average support). Thus, the coefficients β represent the treatment effect of the norm interventions in the respective regions. The constant α represents the mean support in the control group. x_i is a vector of control variables. It includes socio-demographic characteristics (gender (two dummy variables representing female and diverse with male being the omitted category), age (indicator variable for above-median values), income (indicator variable for above-median values), education level (indicator variable for tertiary education)), NUTS2 regional fixed effects and survey week fixed effects. Regional fixed effects and survey week fixed effects control for omitted variable bias that is specific to regions or the interview time. Standard errors are robust.

Tables

	Treatment		Control		
	Mean	SD	Mean	SD	p-value
Age	49.61	(15.09)	48.50	(16.34)	0.577
Female	0.51	(0.50)	0.39	(0.49)	0.010
Diverse	0.00	(0.04)	0.00	(0.00)	0.669
Income	2963.46	(1706.94)	2892.43	(1895.02)	0.047
Tertiary education	0.30	(0.46)	0.35	(0.48)	0.292
Observations	7191		109		

Table S1: Descriptive statistics and randomization check for survey 1. "Age" is the age of the respondent ranging from 18 to 90 years. "Female" is coded as 1 if the respondent was female and 0 otherwise. "Diverse" is coded as 1 if the respondent was of non-binary gender and 0 otherwise. "Income" is coded as the mean income of the income section the respondent selected to be in. "Tertiary education" is coded as 1 if the respondent has at least a university degree and 0 otherwise. The sample size for education is reduced due to 3 people not answering the question on educational level. The last column shows p-values for the null hypothesis of perfect randomization (χ^2-tests).

Dependent variable:	Actual support		Estimated support		Misperception error		Misperception error	
	(1)	(2)	(3)	(4)	(5)	(6)	(7)	(8)
	Control	Treat.	Control	Treat.	Control	Treat.	Control	Treat.
Constant	3.635***	3.866***	3.603***	3.744***	-0.161	-0.183***	-1.030	-0.691***
	(0.206)	(0.053)	(0.227)	(0.039)	(0.232)	(0.039)	(0.627)	(0.073)
High climate goals	-0.028	-0.247***	-0.037	-0.285***	-0.009	-0.038***	-0.009	-0.038***
	(0.026)	(0.010)	(0.045)	(0.010)	(0.045)	(0.009)	(0.046)	(0.009)
Personal burden of COVID-19							0.007	0.026*
							(0.128)	(0.012)
Finanical impact of COVID-19							-0.002	-0.005
							(0.089)	(0.010)
Impact of recent flood event							-0.153	0.000
							(0.092)	(0.010)
Belief in climate change							0.263+	0.198***
							(0.148)	(0.015)
Implement. of climate protection in EU							-0.250	0.070***
							(0.163)	(0.015)
Implement. of climate protection in Germany							0.340+	0.037*
							(0.185)	(0.017)
Implement. of climate protection in region							0.189	0.069***
							(0.194)	(0.017)
Trust in climate friendly companies							-0.006	0.059***
							(0.094)	(0.010)
Trust in scientists							0.081	0.025
							(0.104)	(0.015)
Trust in supranational institutions							0.087	0.007
							(0.174)	(0.013)
Trust in national institutions							-0.139	-0.003
							(0.136)	(0.011)
Socio-economic controls	Yes	Yes	Yes	Yes	Yes	Yes	Yes	Yes
Survey week FE	Yes	Yes	Yes	Yes	Yes	Yes	Yes	Yes
Nuts2 FE	Yes	Yes	Yes	Yes	Yes	Yes	Yes	Yes
R^2	0.412	0.039	0.424	0.059	0.374	0.012	0.530	0.125
Observations	218	14376	218	14376	218	14376	218	14376

Table S2: Effect of high climate goals on public support for climate policies. Clustered standard errors in parentheses (NUTS2). *** p<0.001, ** p<0.01, * p<0.05, + p<0.1. Columns 1 and 2 show results for actual support as dependent variable, columns 3 and 4 for estimated support and columns 5 to 8 for the misperception error (estimated support - actual support). Both actual and estimated support are measured on a 5 point-scale ranging from 1 to 5 (completely oppose to completely support with neutral option). The coefficients of the explanatory variables are standardized (z-score). They can therefore be interpreted as the difference in support rate associated with a one standard deviation change in the explanatory variable. Depending on the column, specifications include the following control variables: gender, age (median), income (median), education level (tertiary), survey week fixed effects, subnational region fixed effects. The sample size is reduced due to 3 people not answering the question on educational level. Note that the low climate goals scenario was repeated in the control group.

	Control		National norm		Regional norm		Total sample		
	Mean	SD	Mean	SD	Mean	SD	Mean	SD	p-value
Age	50.29	(15.64)	49.93	(15.56)	49.95	(15.48)	50.05	(15.56)	0.815
Female	0.48	(0.50)	0.46	(0.50)	0.48	(0.50)	0.48	(0.50)	0.271
Diverse	0.00	(0.05)	0.00	(0.04)	0.00	(0.05)	0.00	(0.05)	0.908
Income	2738.94	(1529.65)	2832.84	(1717.99)	2835.49	(1616.70)	2803.05	(1624.69)	0.212
Tertiary education	0.30	(0.46)	0.31	(0.46)	0.30	(0.46)	0.30	(0.46)	0.732
Observations	1554		1591		1582		4727		

Table S3: Descriptive statistics and randomization check for survey 2. "Age" is the age of the respondent ranging from 18 to 90 years. "Female" is coded as 1 if the respondent was female and 0 otherwise. "Diverse" is coded as 1 if the respondent was of non-binary gender and 0 otherwise. "Income" is coded as the mean income of the income section the respondent selected to be in. "Tertiary education" is coded as 1 if the respondent has at least a university degree and 0 otherwise. The sample size for income and education is reduced due to 336 people not answering the question on income and 12 people not answering the question on educational level. The last column shows p-values for the null hypothesis of perfect randomization (χ^2-tests).

national norm x below average	0.110+	0.102	0.105*
	(0.066)	(0.069)	(0.052)
regional norm x below average	-0.146*	-0.154*	-0.117*
	(0.068)	(0.071)	(0.053)
national norm x at average	-0.035	-0.045	-0.113+
	(0.076)	(0.078)	(0.064)
regional norm x at average	0.047	0.047	-0.040
	(0.076)	(0.078)	(0.062)
national norm x above average	0.157**	0.140*	0.112*
	(0.059)	(0.061)	(0.049)
regional norm x above average	0.080	0.061	0.067
	(0.061)	(0.063)	(0.050)
Personal burden of COVID-19			-0.050**
			(0.015)
Finanical impact of COVID-19			0.012
			(0.015)
Impact of recent flood event			-0.006
			(0.018)
Belief in climate change			0.626***
			(0.019)
Implement. of climate protection in region			-0.055*
			(0.025)
Implement. of climate protection in Germany			-0.053+
			(0.027)
Implement. of climate protection in EU			0.046+
			(0.026)
Trust in climate friendly companies			0.048*
			(0.022)
Trust in scientists			0.042*
			(0.021)
Trust in supranational institutions			0.157***
			(0.024)
Trust in national institutions			0.023
			(0.024)
Constant	3.417***	3.718***	3.639***
	(0.061)	(0.073)	(0.100)
Socio-economic controls	No	Yes	Yes
Survey week FE	Yes	Yes	Yes
Nuts2 FE	Yes	Yes	Yes
R^2	0.038	0.058	0.515
Observations	4727	4387	3869

Table S 4: Treatment effect of social norms on support. OLS regression. The specification shows interactions between treatments and subnational regions. Robust standard errors are in parentheses. *** p<0.001, ** p<0.01, * p<0.05, + p<0.10. Support is measured on a 5 point-scale ranging from 1 to 5 (completely oppose to completely support with neutral option). Specifications include the following control variables: gender, age (median), education level (tertiary), survey week fixed effects, subnational region fixed effects.

Additional Figures

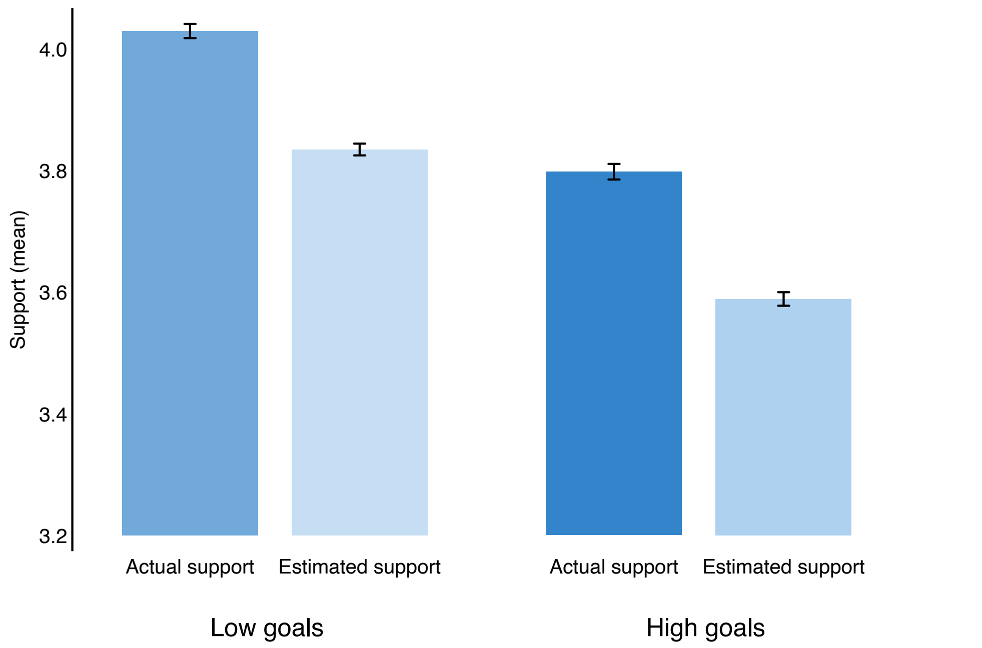

Figure S1: Mean support for climate policies under low and high climate goals for injunctive norm. The figure shows mean actual and estimated support for the injunctive norm, i.e. the norm based on peoples' perception of peoples' actual behavior, once for the low and once for the high climate goals. Both actual and estimated support are measured on a 5-point scale (completely oppose to completely support with neutral option). Bars indicate standard error of the mean. Observations = 7,173.

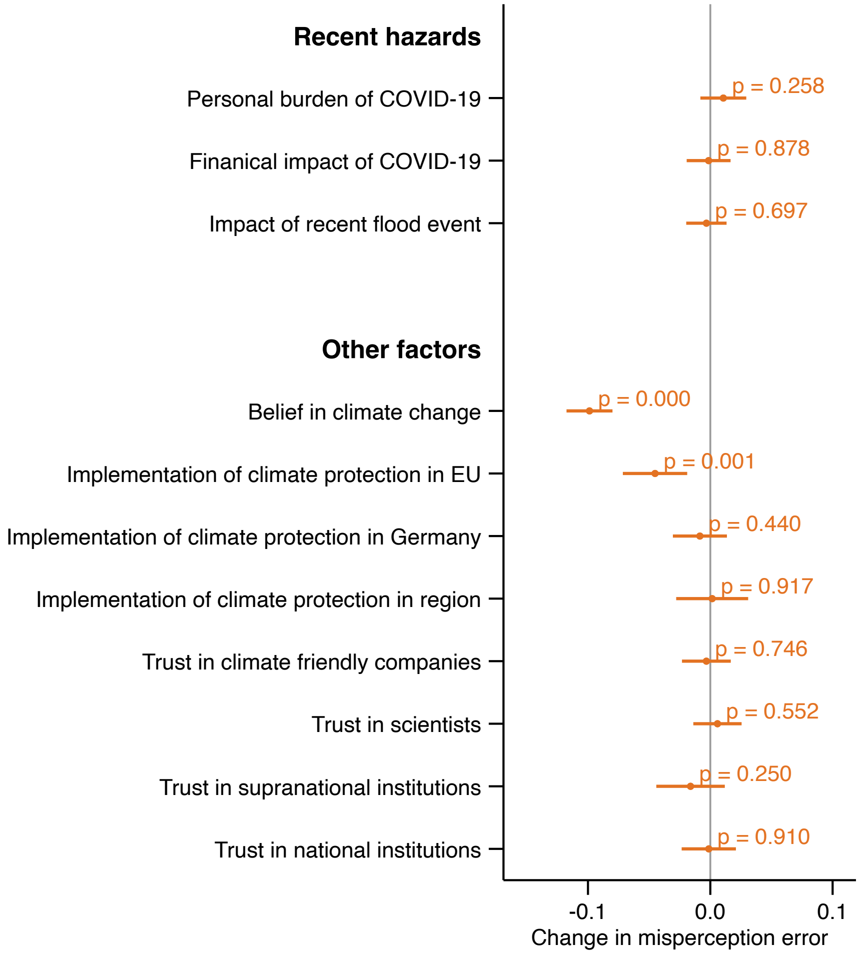

Figure S2: Correlations with individual factors for high climate goals. The figure plots coefficients based on an OLS regression. The specification is based on equation (2). The dependent variable is the absolute value of the misperception error (estimated support - actual support). Both actual and estimated support are measured on a 5 point-scale ranging from 1 to 5 (completely oppose to completely support with neutral option). The regression includes the following control variables: gender, age (median), income (median), education level (tertiary), survey week fixed effects, subnational region fixed effects. The coefficients of the explanatory variables are standardized (z-score). They can therefore be interpreted as the difference in support rate associated with a one standard deviation change in the explanatory variable. Error bars indicate 95% confidence intervals obtained from standard errors that are clustered at the sub-national level. The sample size is reduced due to 3 people not answering the question on educational level. Observations = 7,188.

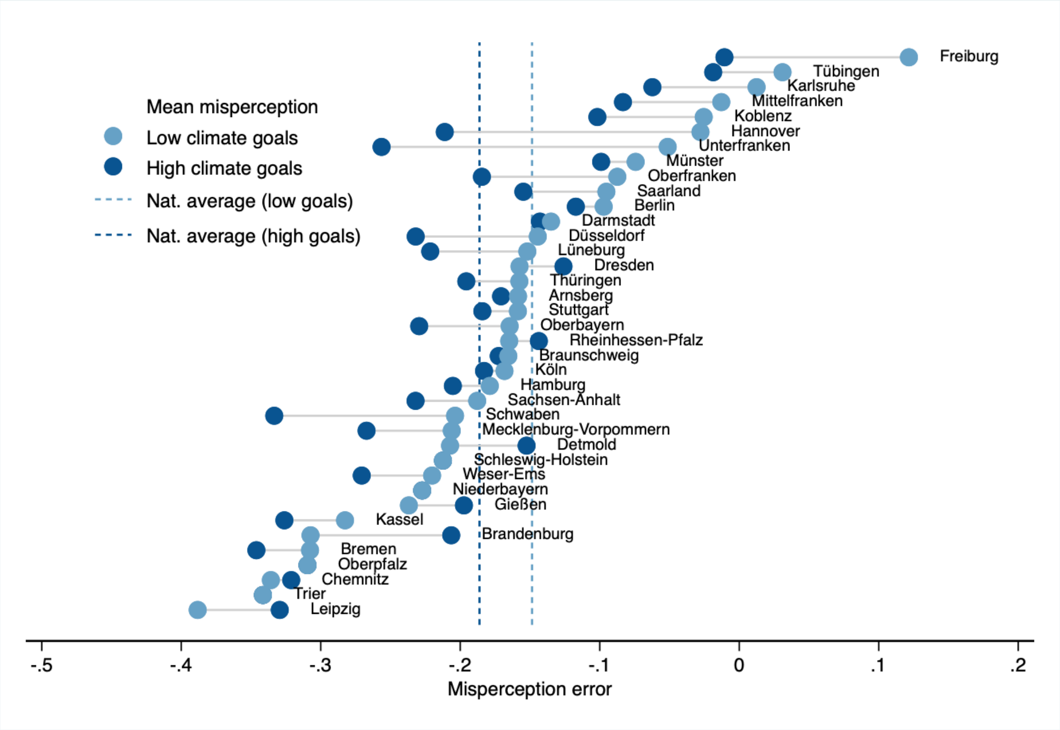

Figure S3: Regional heterogeneity in misperception error for low and high climate goals. The figure shows the mean misperception error for each of the 38 NUTS regions. Here, the misperception error is defined as the difference between mean actual and estimated support for EU climate policies within each respective NUTS2 region. Both actual and estimated support are measured on a 5-point scale (completely oppose to completely support with neutral option). The dashed lines represents the national mean of actual support for low and high goals, respectively. Observations = 7,191.

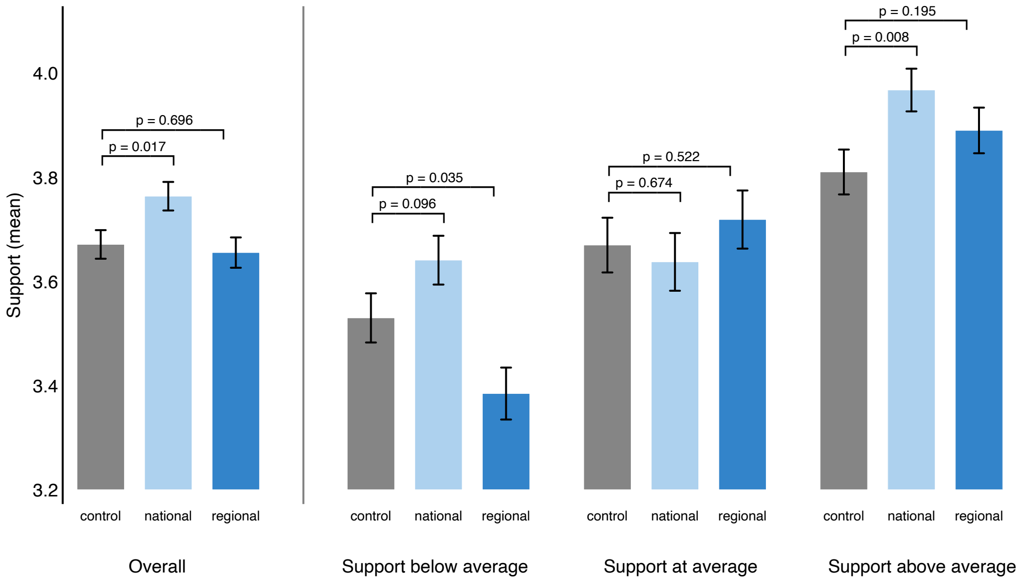

Figure S4: Support across treatments and levels of support in regions. The figure shows results for each of the two treatment groups and the control group. The national treatment group received information on a social norm based on the national average support of EU climate policies while the regional treatment group received this information based on respective regional average support. The control group did not receive any information on a social norm. The experiment was conducted in three regions that had a mean support below the national average, two regions with mean support at the national average and three regions with support above the national average. Error bars indicate standard errors of the mean. The p-values are based on two-sided t-tests comparing the control group to the national or regional treatment group, respectively. Observations: overall = 4,727 ; control = 1,554; national = 1,591; regional = 1,582.

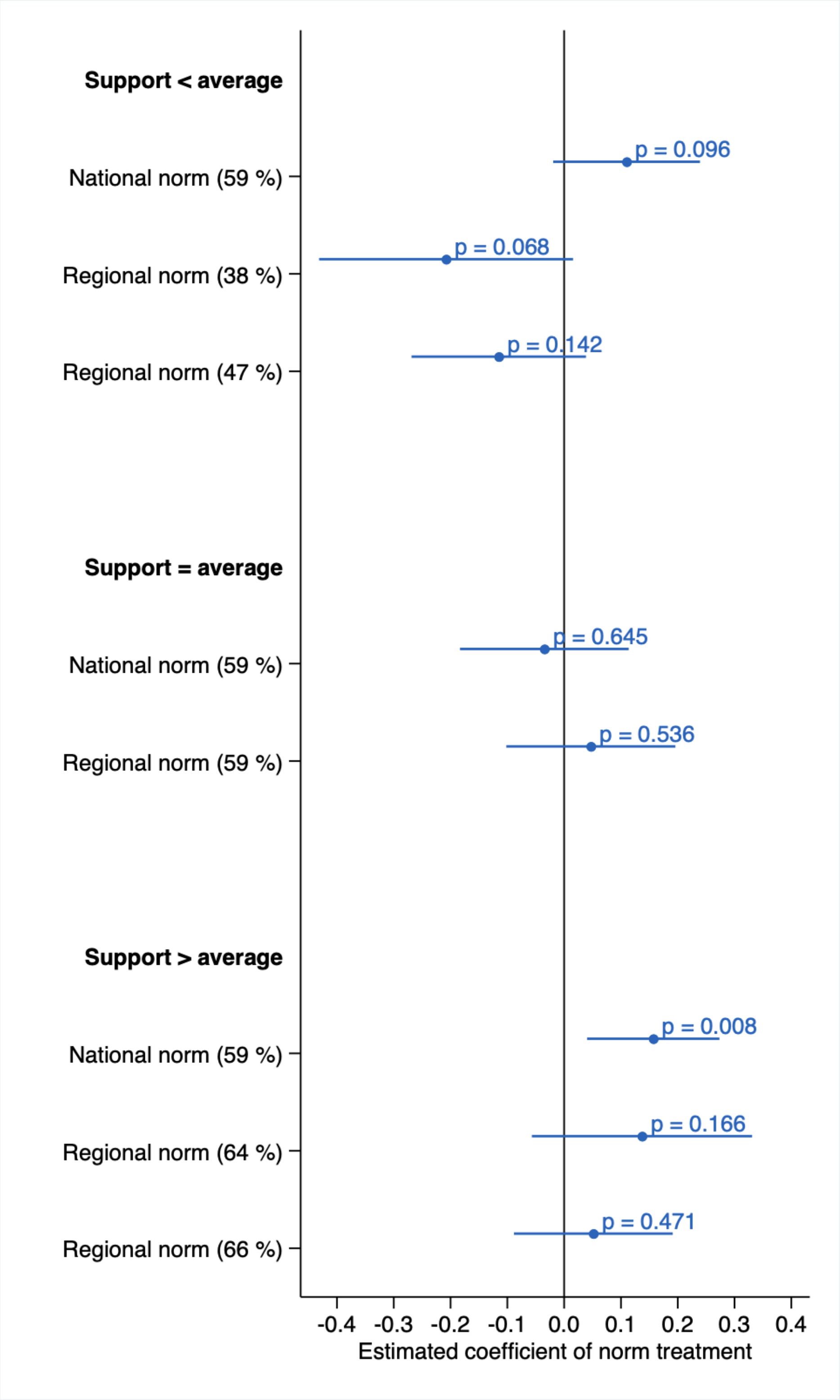

Figure S5: Treatment effect of social norms on support within support regions. OLS regression. The specification shows interactions between treatments and subnational regions. Brackets include information on norm message (% of people supporting high goals in corresponding regions). Robust standard errors are in parentheses. Support is measured on a 5 point-scale ranging from 1 to 5 (completely oppose to completely support with neutral option). Specification is based on column 1 of Table S4.
