# Supplementary figures and images for: Regional variation in social norm nudges
Source: Sci Rep. 2024 Jul 22;14:16773. doi: 10.1038/s41598-024-65765-z (PMC11263545; doi:10.1038/s41598-024-65765-z)

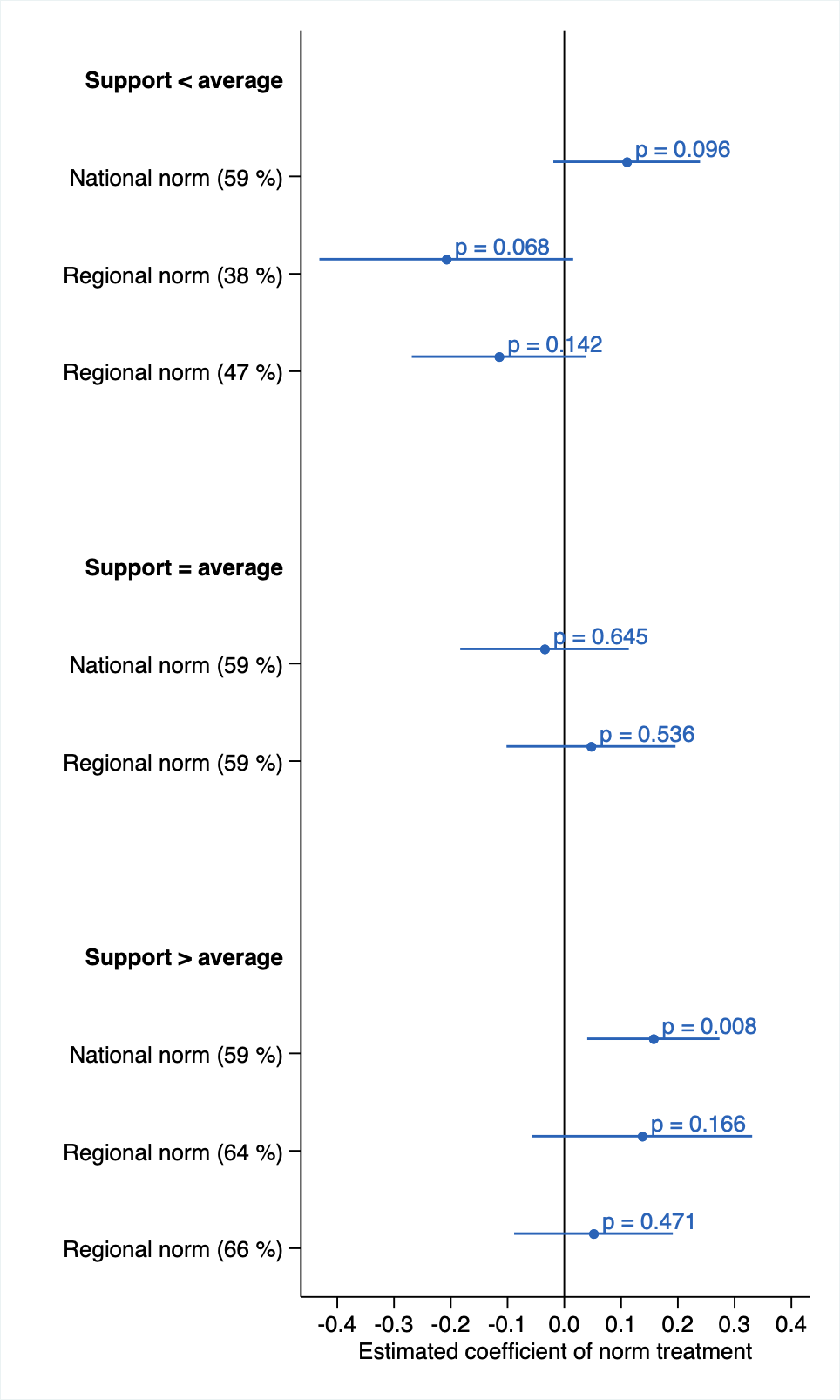

Supplement: Supplementary file 2 — Supplementary Information 2. [file 41598_2024_65765_MOESM2_ESM.zip › Replication2/figures/coefplot_norms_allinterventions.png]

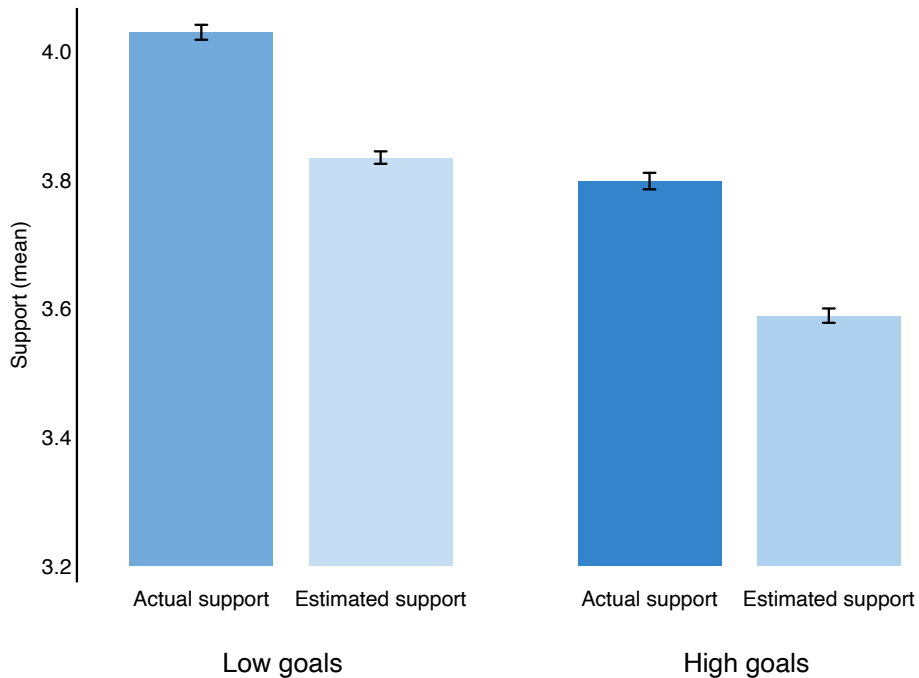

Supplement: Supplementary file 2 — Supplementary Information 2. [file 41598_2024_65765_MOESM2_ESM.zip › Replication2/figures/norms_bar_misperception_PM_pn.pdf]

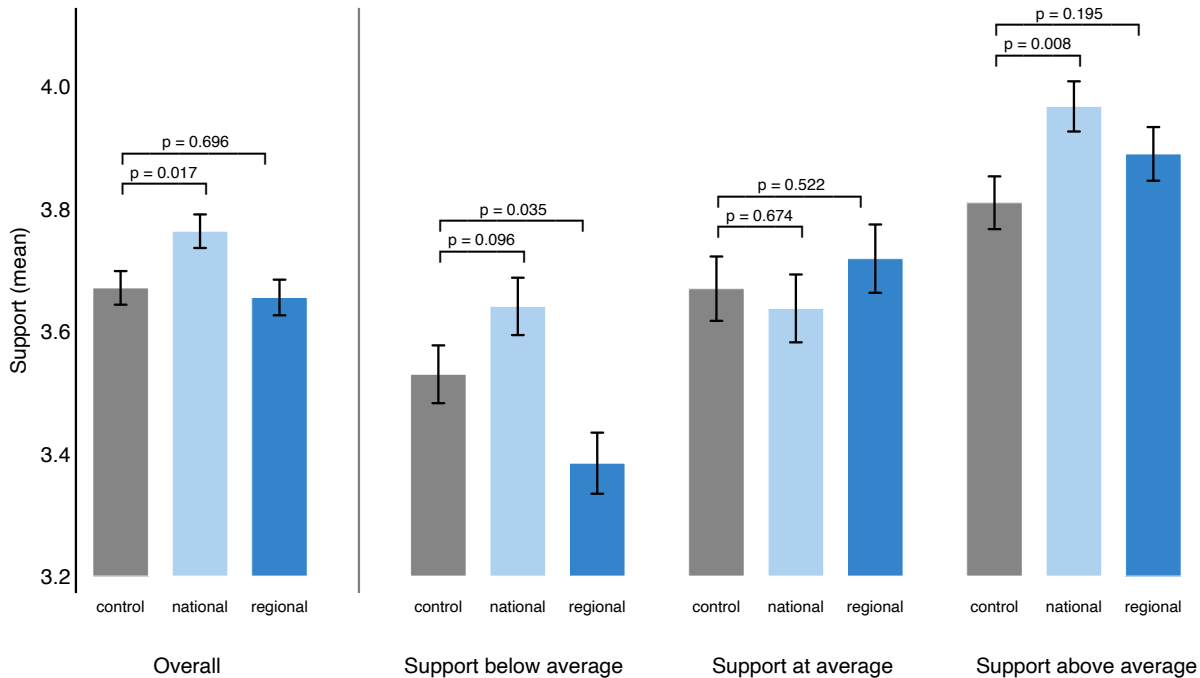

Supplement: Supplementary file 2 — Supplementary Information 2. [file 41598_2024_65765_MOESM2_ESM.zip › Replication2/figures/norms_survey_bar_support_mean.pdf]

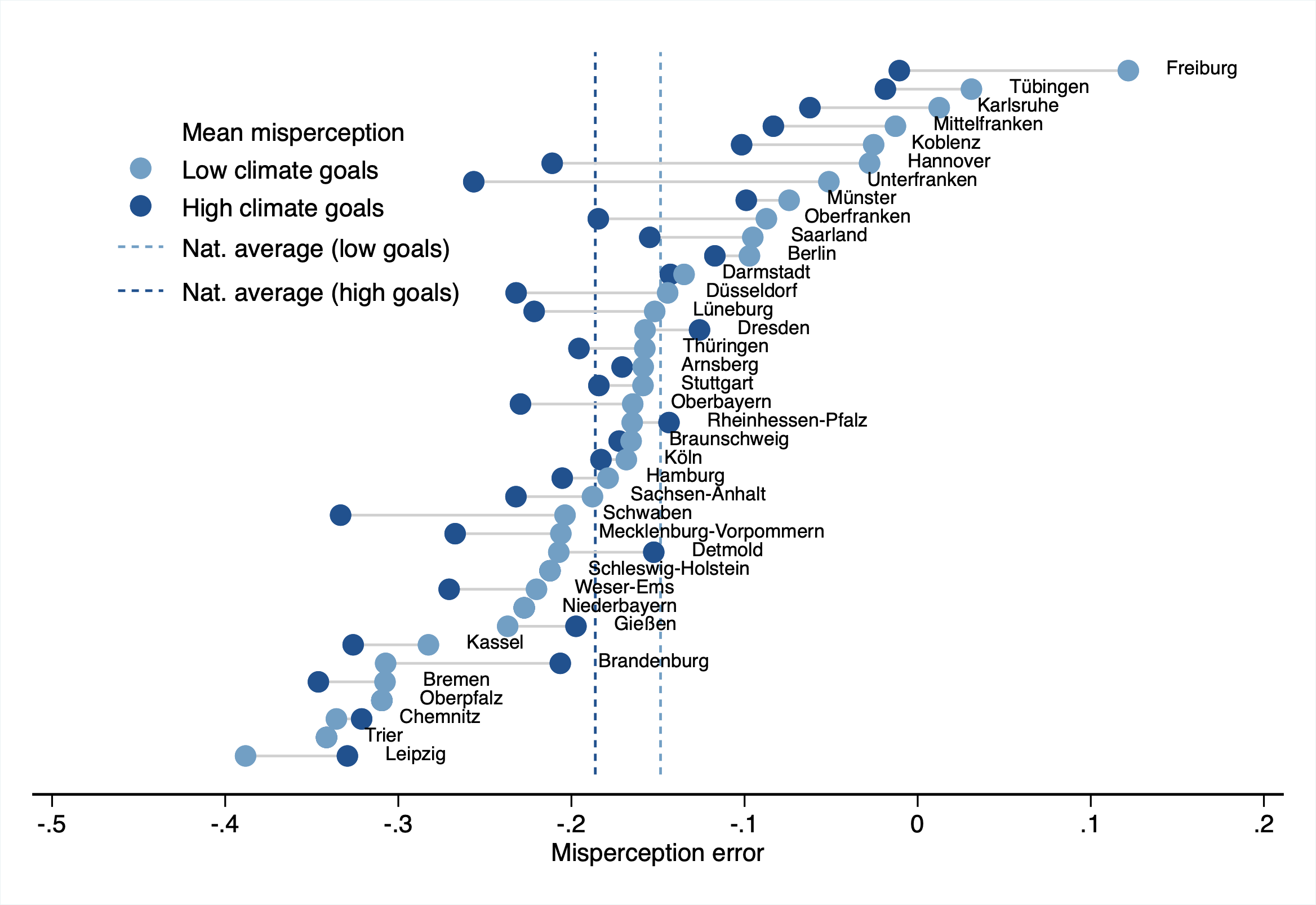

Supplement: Supplementary file 2 — Supplementary Information 2. [file 41598_2024_65765_MOESM2_ESM.zip › Replication2/figures/norms_regvar_pb_misperception.png]

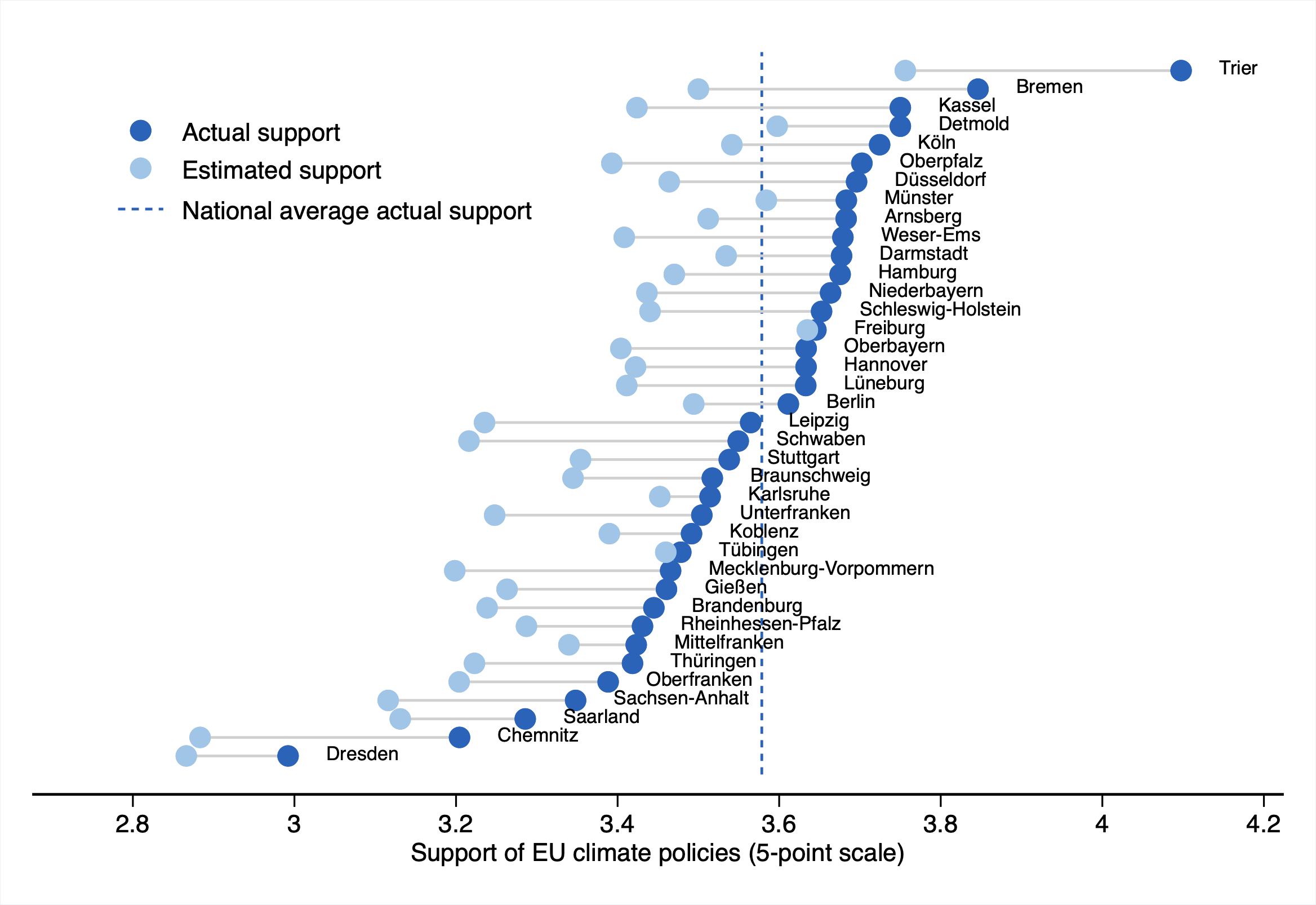

Supplement: Supplementary file 2 — Supplementary Information 2. [file 41598_2024_65765_MOESM2_ESM.zip › Replication2/figures/norms_regvar_pb_m.png]
