# Supplementary material for: Regional variation in social norm nudges
Source: Sci Rep. 2024 Jul 22;14:16773. doi: 10.1038/s41598-024-65765-z (PMC11263545; doi:10.1038/s41598-024-65765-z)
Supplement: Supplementary file 2 — Supplementary Information 2. [file norms_coefindfact_abs_pm.pdf › Replication2/figures/norms_coefindfact_abs_pm.pdf]

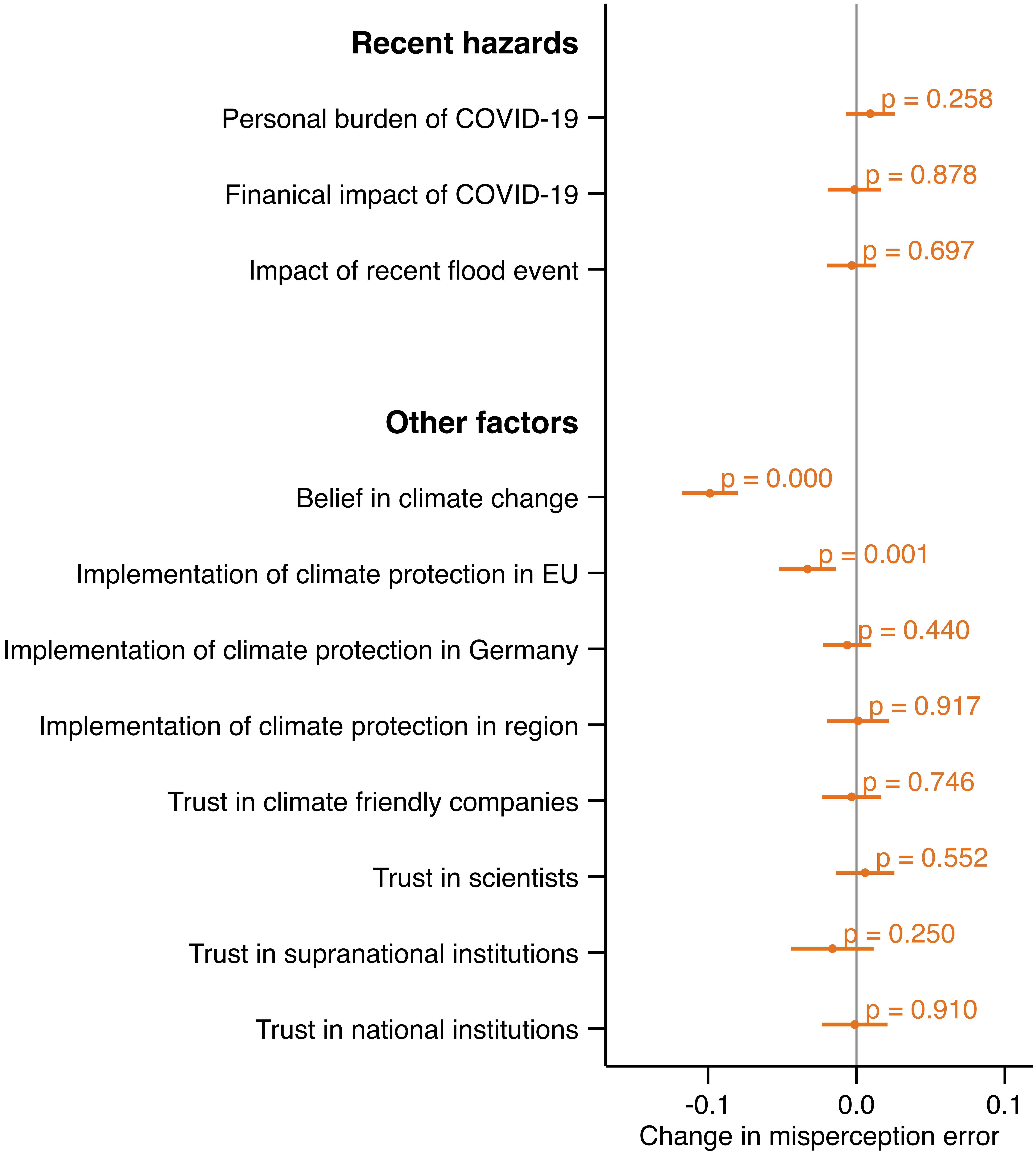

Recent hazards
Personal burden of COVID-19
p = 0.258
Finanical impact of COVID-19
p = 0.878
Impact of recent flood event
p = 0.697
Other factors
Belief in climate change
p = 0.000
Implementation of climate protection in EU
p = 0.001
Implementation of climate protection in Germany
p = 0.440
Implementation of climate protection in region
p = 0.917
Trust in climate friendly companies
p = 0.746
Trust in scientists
p = 0.552
Trust in supranational institutions
p = 0.250
Trust in national institutions
p = 0.910
-0.1
0.0
0.1
Change in misperception error
